# Stress–Strain Engineering with Lithium Solid Solution/Lithium Alloy Composite Anodes for Stable Lithium‐Metal Batteries

**DOI:** 10.1002/advs.202515264

**Published:** 2025-09-23

**Authors:** Junjie Fu, Xiancheng Wang, Xiangrui Duan, Zihe Chen, Tiancheng Dong, Hengtao Shen, Chunhao Li, Renming Zhan, Yangtao Ou, Shiyu Liu, Yongming Sun

**Affiliations:** ^1^ Wuhan National Laboratory for Optoelectronics Huazhong University of Science and Technology Wuhan 430074 China

**Keywords:** Li alloy, Li metal pouch cells, Li solid solution, operando spatial stress measurement, stress−strain

## Abstract

High‐performance thin lithium (Li) metal anodes are indispensable for high‐energy‐density Li metal batteries, yet their electrochemical‐mechanical instability precipitates rapid failure. Uncontrolled lithium plating, accompanied by severe side reactions and the formation of a porous, non‐compact deposit, swells the electrode and generates destructive stress. An in situ investigation of stress, strain, and their spatial distributions during charge/discharge cycling identifies the coupled volume variation and stress–strain evolution as an operando descriptor of degradation and underpins the rational design of a dual‐phase Li−Ag solid‐solution/Li_22_Sn_5_ alloy composite anode. Ag in Li−Ag solid solution enhances the affinity with Li^+^ for uniform Li plating, whereas a rigid, ion‐permeable Li_22_Sn_5_ scaffold confines volume expansion. LiNi_0.6_Co_0.2_MnO_0.2_ (NCM622)||Li−Ag/Li_22_Sn_5_ pouch cell exhibits a markedly lower internal‐pressure rise during cycling than NCM622||Li counterpart under identical conditions. Specifically, the latter shows a 58% higher pressure increase than the NCM622||Li−Ag/Li_22_Sn_5_ pouch cell after charging from 0% to 100% SOC in the 5th cycle. The 1.3 Ah laminated NCM622||Li−Ag/Li_22_Sn_5_ (40 µm‐thick anode) pouch cell retains 87% capacity over 100 cycles when operating at an industry‐relevant N/P ratio of 1.5 and electrolyte budget of 2.6 g Ah^−1^. Mechanical‐electrochemical coupling of Li metal anode design, therefore, offers a practical route to long‐lifespan Li metal batteries.

## Graphical abstract

A Li−Ag/Li_22_Sn_5_ composite is explored as a Li‐metal anode. The Ag‐rich solid solution guides uniform Li⁺ deposition, while the Li_22_Sn_5_ scaffold confines volume expansion. Operando stress mapping reveals mitigated stress fluctuations and homogenized stress distribution, leading to enhanced electrochemical‐mechanical stability and prolonged cycling life.

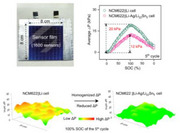

## 1 Introduction

Li metal batteries have emerged as a highly promising successor to conventional Li‐ion batteries, primarily due to their exceptionally high theoretical energy density.^[^ ^1^, ^2^, ^3^ ^]^ Nevertheless, their practical implementation faces substantial obstacles, especially concerning the cyclic durability and operational safety of Li metal anodes. These anodes, known for their ultralow electrochemical potential (−3.04 V vs standard hydrogen electrode) and unmatched theoretical capacity (3860 mAh g^−1^), experience substantial volume changes and high chemical reactivity during charge/discharge cycles, leading to rapid mechanical and electrochemical failure.^[^ ^4^, ^5^ ^]^ Inherent volume fluctuations in Li metal anodes induce significant mechanical stress, while uneven Li plating generates loose, porous deposits, which exacerbate strain accumulation and accelerate undesirable side reactions between active Li and the electrolyte.^[^ ^6^, ^7^, ^8^ ^]^ Such coupled mechanical and chemical degradation adversely impacts the integrity and stability of the Li metal anodes, ultimately compromising the overall performance and longevity of the battery.

The stress and strain conditions within the anode and the overall battery system are critical indicators of Li metal battery performance, directly reflecting battery stability and safety.^[^ ^9^, ^10^ ^]^ Minimizing volume change during charge/discharge cycling and preserving a uniform electrode morphology stabilizes the anode and suppresses side reactions between the Li metal anode and the electrolyte, thereby extending the battery lifespan.^[^ ^11^, ^12^, ^13^ ^]^ Research to date has centered on electrolyte optimization, composite Li architectures, and interface engineering to boost anode performance.^[^ ^14^, ^15^, ^16^, ^17^, ^18^, ^19^, ^20^, ^21^, ^22^, ^23^ ^]^ Composite Li metal designs, encompassing Li alloys, polymers, and carbon‐based hosts, are widely recognized as effective regulators of volume change.^[^ ^24^, ^25^, ^26^, ^27^, ^28^, ^29^, ^30^ ^]^ Composite Li metal anodes have long been recognized for their ability to homogenize and stabilize Li plating/stripping and to mitigate strain and stress accumulation during cycling. However, definitive validation through in situ mechanical measurements has remained absent. Consequently, there is a critical need to investigate these strategies from the perspective of coupled electrochemical and mechanical stability. Clarifying how stress develops, how interfaces degrade, and how structural integrity is lost will inform rational materials design. To this end, the development of advanced materials design capable of simultaneously mitigating volume fluctuations during cycling, regulating electrochemical behavior, and suppressing parasitic reactions at the Li/electrolyte interface is imperative for enabling stable and high‐performance Li metal anodes.

In this study, we introduce a composite anode consisting of a Li solid solution and Li intermetallic compounds. The Li intermetallic compounds serve as a rigid framework for Li^+^ conduction and provide mechanical support,^[^ ^31^, ^32^, ^33^, ^34^, ^35^, ^36^, ^37^ ^]^ while the metallic species dissolved in the Li solid solution acts as a regulator for in situ Li plating.^[^ ^38^, ^39^, ^40^, ^41^, ^42^, ^43^ ^]^ By integrating these two phases, the anode sustains low global strain and stress throughout prolonged plating/stripping, which translates into markedly enhanced cycling stability. Experimentally, a Li−Ag/Li_22_Sn_5_ composite foil was prepared as a model system for verification via a two‐step process under inert atmosphere. The Li−Ag/Li_22_Sn_5_ composite anode effectively maintains low interfacial strain and stress, wherein the Li_22_Sn_5_ phase acts as a stable skeleton providing ion‐transport pathways and mechanical support, while the Li−Ag solid solution facilitates uniform lithium deposition. Operando spatial‐stress mapping confirms that the composite maintains minimal strain accumulation during cycling. When paired with a commercial NCM622 cathode, the pouch cell incorporating the Li−Ag/Li_22_Sn_5_ anode exhibited a substantially lower pressure increase (ΔP) during the charge/discharge process relative to that observed for the cell with a Li anode, the latter of which exhibited a 58% increase after charging from 0% to 100% SOC in the 5th cycle. A 1.3 Ah laminated pouch cell employing a NCM622 cathode and a Li−Ag/Li_22_Sn_5_ (40 µm) anode showed stable cycling performance for over 100 cycles, with a capacity retention of 87% at a low N/P ratio of 1.5 and an electrolyte/capacity (E/C) ratio of 2.6 g Ah^−1^. By coupling a Li solid solution with a Li^+^‐conductive intermetallic framework, the composite anode simultaneously regulates mechanical stress and suppresses parasitic reactions, opening a viable pathway toward Li metal batteries with greatly extended lifespans.

## 2 Results and Discussion

### 2.1 Stress‐Mitigation Mechanism and Operando Quantification of a Li−Ag/Li_22_Sn_5_ Composite Anode

**Figure 1:**
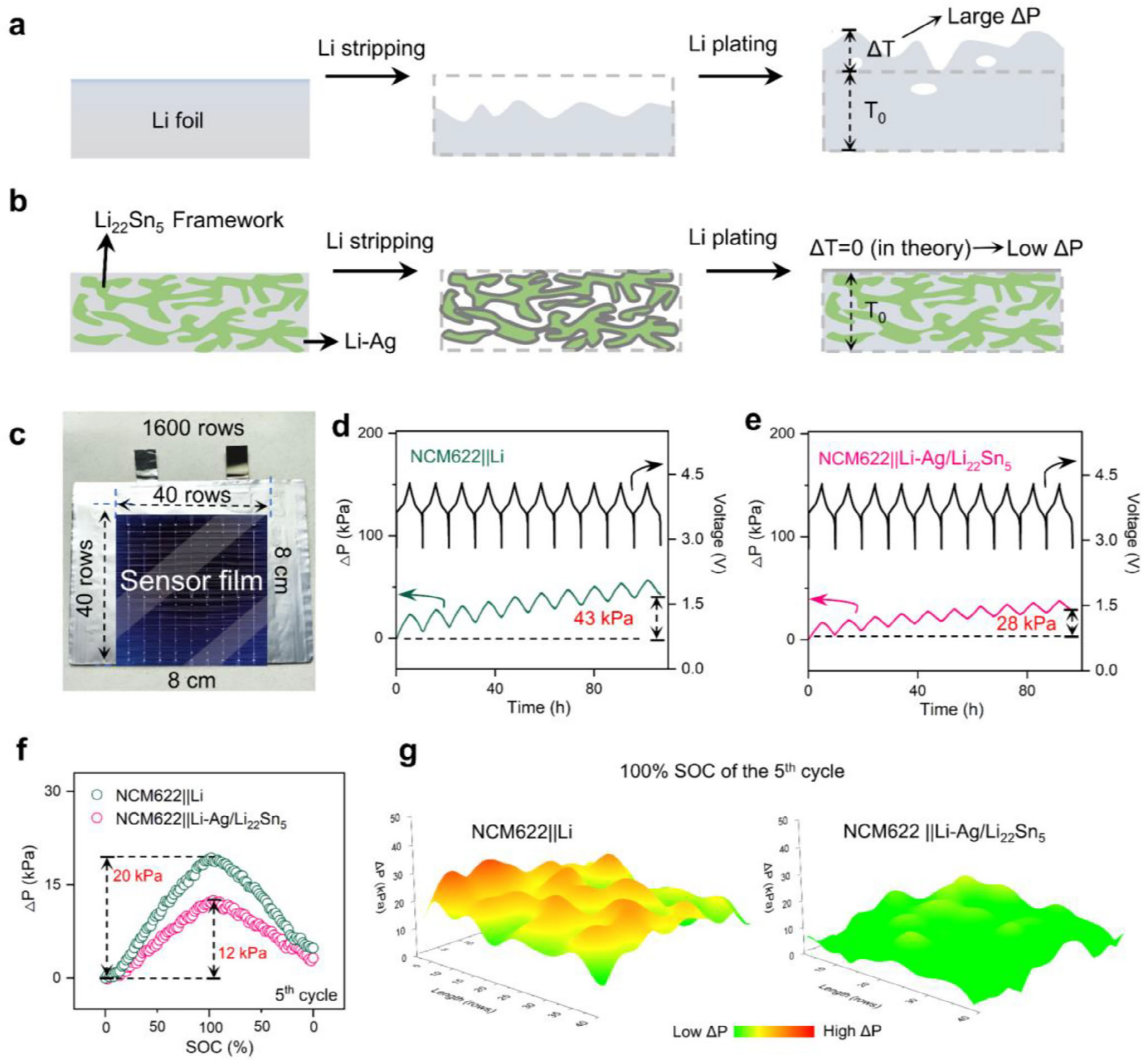
Schematic of Li stripping/plating on a) Li foil and b) Li−Ag/Li_22_Sn_5_ composite foil. c) Photograph of the as‐prepared NMC622||Li−Ag/Li_22_Sn_5_ laminated pouch cell with an 8 × 8 cm tested zone (electrode region). The inset shows a schematic of the force‐sensor film with an ordered 40 × 40 array of sensing nodes (1600 in total). d,e) Evolution of self‐generated pressures during charge/discharge for the first 10 cycles of NCM622||Li and NCM622||Li−Ag/Li_22_Sn_5_ pouch cells, respectively. f) Pressure variation during the 5th cycle for the same cells. g) 3D pressure maps at 100% state of charge in the 5th cycle for NCM622||Li and NCM622||Li−Ag/Li_22_Sn_5_ pouch cells.

During the operando spatial stress measurement setup operation, pouch cells experience mechanical confinement under fixed constraints. The thickness deviations (ΔT) induced by Li plating, reflecting the degree of thickness changes throughout the electrode area, inevitably generate corresponding pressure fluctuations (ΔP) against the imposed constraints. As depicted in **Figure** **1**a, a pure Li electrode demonstrates non‐uniform electrochemical dissolution and deposition behaviors, resulting in a porous and heterogeneous structure of the deposited Li layer. This disparity leads to uneven thickness growth at distinct locations on the Li metal electrode, causing significant and irregular stress buildup across the electrode. In contrast, the mixed electronic/ionic conductive Li_22_Sn_5_ framework of the Li−Ag/Li_22_Sn_5_ electrode confines Li within the framework for electrochemical deposition and dissolution, promoting a more uniform Li deposition/dissolution process. This aids in maintaining a low and consistent strain distribution across the electrode during cycling (Figure 1b). Moreover, Ag in the Li−Ag solid solution synergistically densifies the Li deposit, stabilizing the Li/electrolyte interface and suppressing side reactions.^[^ ^24^ ^]^ Consequently, spatial and temporal variations in electrode thickness are diminished, yielding uniform thickness evolution. The employed force‐sensor film enables real‐time measurement of pressure fluctuations associated with the Li dissolution and deposition processes within the electrode, capturing both temporal and spatial dimensions.

As depicted in Figure 1c, the force‐sensor film was applied to practical pouch cells (with active area of 8 × 8 cm) featuring an organized array of 1600 sensor nodes arranged in 40 × 40 rows. These cells clamped between compliant fixtures to quantify pressure distribution. Figure 1d,e illustrates the progression of internally generated pressures during the initial 10 charge/discharge cycles at 0.2 C (after 0.1 C activation) under an external pressure (P) of ≈300 kPa. Cycling performance of NCM622||Li and NCM622||Li−Ag/Li_22_Sn_5_ pouch cells are displayed in Figure S8 (Supporting Information). In the case of NCM622||Li pouch cells employing a Li electrode, the pressure increase (ΔP) value reached 23 kPa during the first charging cycle and rose to 43 kPa after 10 cycles (Figure 1d). Conversely, for the NCM622||Li−Ag/Li_22_Sn_5_ cell, the corresponding ΔP values were only 17 and 28 kPa, respectively (Figure 1e). The ΔP values of NCM622||Li and NCM622||Li−Ag/Li_22_Sn_5_ during the 5th charging cycle from 0% state of charge (SOC) to 100% SOC were ≈20 and 12 kPa, respectively, with the former being 58% higher than the latter. The large ΔP values observed in the NCM622||Li cell are attributed to the disordered and heterogeneous Li deposition during the charging process. The distribution histogram of ΔP calculated from 1600 data points after charging in the 5th cycle is also shown in Figure S9 (Supporting Information). A more concentrated ΔP distribution for the NCM622||Li−Ag/Li_22_Sn_5_ cell indicates a more uniform and lower pressure compared to the NCM622||Li cell. These characteristics are clearly evident in the 3D topography of pressure differentials after charging from 0% SOC to 100% SOC in the 5th cycle (Figure 1g). A comparison between NCM622||Li and NCM622||Li−Ag/Li_22_Sn_5_ cells reveals a heterogeneous stress distribution in the former. To validate the general applicability of the Li−Ag/Li_22_Sn_5_ electrode, cells with sulfurized polyacrylonitrile (SPAN) cathode were evaluated to highlight the role of lithium solid solution/lithium alloy composite anodes in enhancing the stability of Li metal batteries. Similar behavior is confirmed in Figures S10–S12 (Supporting Information), where Li−Ag/Li_22_Sn_5_ anodes paired with SPAN exhibited lower stress and strain under an external pressure (P) of ≈1 MPa. Collectively, the composite Li metal anode exhibited minimal, uniform stress variation during Li plating/stripping, resulting in small, consistent thickness changes. This improvement enhances electrochemical‐mechanical stability during cycling, thereby extending battery longevity and improving safety.

### 2.2 Electrochemical Kinetics and Cycling Performance of the Li−Ag/Li_22_Sn_5_ Composite Anode

**Figure 2:**
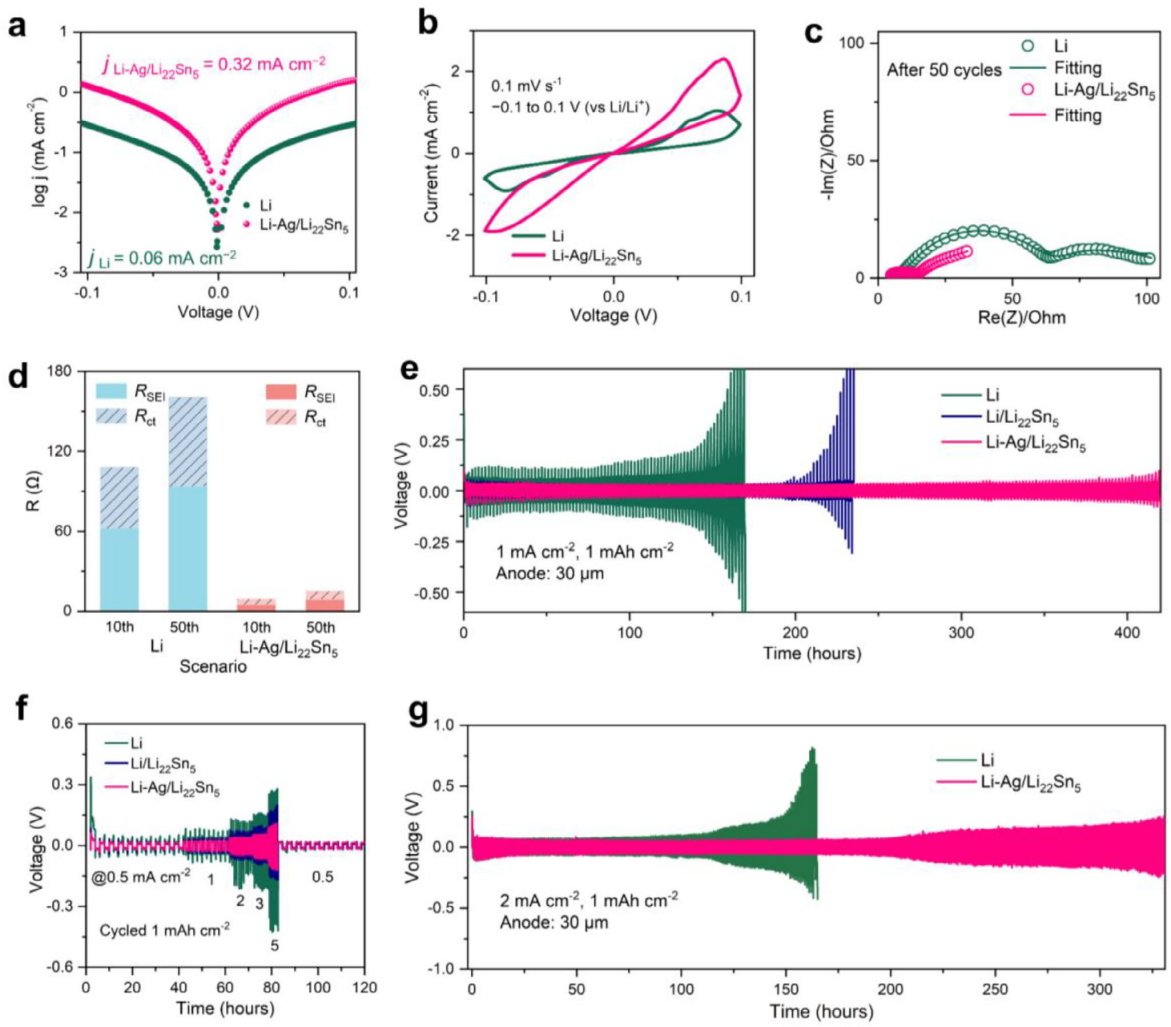
a) Tafel plots for symmetric cells with Li and Li−Ag/Li_22_Sn_5_ electrodes. b) Cyclic‐voltammetry curves (0.1 mV s^−1^) of the same cells. c) Nyquist plots after 50 cycles at 1 mA cm^−2^ (cut‐off 1 mAh cm^−2^). d) Comparison of R_SEI_ and R_ct_ values for Li and Li−Ag/Li_22_Sn_5_ electrodes. e) Voltage profiles of symmetric cells employing Li, Li/Li_22_Sn_5_ and Li−Ag/Li_22_Sn_5_ electrodes (with the anode thickness of 30 µm) at 1 mA cm^−2^, 1 mAh cm^−2^. f) Overpotential as a function of current density (0.5 to 5 mA cm^−2^, 1 mAh cm^−2^) for the three electrodes. g) Voltage profiles at 2 mA cm^−2^, 1 mAh cm^−2^ for Li, and Li−Ag/Li_22_Sn_5_ symmetric cells (with the anode thickness of 30 µm).

To assess the effectiveness of the Li−Ag/Li_22_Sn_5_ composite anode in stabilizing metallic Li electrodes, the electrochemical performance of Li−Ag/Li_22_Sn_5_ and Li symmetric cells was evaluated under various conditions. The exchange current densities (j_0_) were calculated based on linear sweep voltammetry (LSV) measurements. As shown in **Figure** **2**a, the Li−Ag/Li_22_Sn_5_ composite electrode exhibited a significantly higher exchange current density (j_0_) of 0.32 mA cm^−2^, which is an order of magnitude greater than that of the Li electrode (0.06 mA cm^−2^). This marked improvement in Li^+^ transport kinetics at the electrolyte/electrode interface is primarily attributed to the 3D Li_22_Sn_5_ alloy framework within the composite anode. Since the delithiation potential of Li_22_Sn_5_ (≈0.4 V vs Li/Li⁺) is much higher than the dissolution potential of metallic lithium (typically <0.1 V vs Li/Li⁺), the excess metallic lithium in the Li−Ag/Li_22_Sn_5_ electrode prevents delithiation of Li_22_Sn_5_ during cycling. The Li_22_Sn_5_ skeleton not only facilitates efficient ion transport pathways but also provides structural stability during cycling. Further insights into electrochemical reaction kinetics were obtained via cyclic voltammetry (CV) analysis. The composite foil electrode demonstrated substantially enhanced current densities compared to the Li foil electrode in the voltage range from −0.1 to 0.1 V (vs Li/Li^+^) at the scan rate of 0.1 mV s^−1^, indicating accelerated ion transport and electrochemical reactions (Figure 2b). For a comprehensive evaluation of the Li−Ag/Li_22_Sn_5_ composite anode's influence on solid electrolyte interphase (SEI) stability and electrochemical kinetics promotion, Electrochemical impedance spectroscopy (EIS) measurements were carried out using symmetric cells with both composite and Li electrodes under varied conditions. Nyquist plots in Figures 2c and S13 (Supporting Information) correspond to symmetric cells cycled at 1 mA cm^−2^ to 1 mAh cm^−2^ after 10 and 50 cycles. The equivalent circuit models for fitting Li and Li−Ag/Li_22_Sn_5_ are depicted in Figure S14 (Supporting Information). Figure 2d summarizes the comparison, revealing significantly lower SEI resistance (R_SEI_) for the Li−Ag/Li_22_Sn_5_ anode versus the Li electrode. Quantitatively, R_SEI_ values were 5 Ω (10 cycles) and 9 Ω (50 cycles) for the composite, whereas the Li electrode showed 62 and 94 Ω, respectively, under the same conditions. These data underline the composite's ability to stabilize the SEI and lower kinetic barriers.

The electrochemical cycling performance of Li−Ag/Li_22_Sn_5_ and Li electrodes was further investigated using symmetric cells in carbonate electrolyte. As illustrated in Figure 2e, the Li−Ag/Li_22_Sn_5_ composite electrode, with a controlled thickness of 30 µm, demonstrated a significantly extended cycle life exceeding 400 h at 1 mA cm^−2^ and 1 mAh cm^−2^. In contrast, shorter lifetimes of 220 and 170 h were observed for the Li/Li_22_Sn_5_ and Li electrodes, respectively. Notably, the Li−Ag/Li_22_Sn_5_ electrode exhibited a stable and lower potential hysteresis, remaining below 40 mV after 130 h of cycling (Figure S16, Supporting Information). In sharp contrast, the Li electrode displayed pronounced voltage fluctuations and a high potential hysteresis exceeding 150 mV after the same duration. These issues in Li electrodes can be attributed to repetitive SEI fracture and reconstruction, as well as the accumulation of inactive Li during Li plating/stripping cycles. The rate capability of the electrodes was assessed under varying current densities, as shown in Figure 2f. The Li−Ag/Li_22_Sn_5_ composite electrode delivered stable voltage hysteresis values of 12, 20, 39, 53, and 100 mV at current densities of 0.5, 1, 2, 3, and 5 mA cm^−2^, respectively. Comparatively, symmetric cells with the Li/Li_22_Sn_5_ electrode exhibited a higher polarization voltage of 157 mV at 5 mA cm^−2^, while the Li electrode showed a significantly larger overpotential of 267 mV at the same current density. These higher polarization values for Li electrodes are indicative of severe electrochemical kinetic barriers arising during Li plating/stripping processes. To further evaluate the cycling stability of the electrodes at higher current densities, symmetric cells were tested at 2 mA cm^−2^ (Figure 2g). Even under this demanding condition, the Li−Ag/Li_22_Sn_5_ electrode exhibited stable cycling performance with a cycle life exceeding 350 h, coupled with a consistently low overpotential throughout the cycling process. In contrast, the Li electrode suffered from considerably higher overpotentials during the initial charging process and demonstrated premature failure, cycling for only 160 h before complete degradation. We performed a comparative high‐temperature (60 °C) storage test on the Li−Ag/Li_22_Sn_5_ and Li electrodes, during which EIS was used to monitor interfacial resistance. Under identical conditions, after storage for 6 days, the R_SEI_ of the Li symmetric cell reached 655 Ω, while that of the Li−Ag/Li_22_Sn_5_ symmetric cell was only 60 Ω (Figure S17, Supporting Information). These results highlight the superior ability of the Li−Ag/Li_22_Sn_5_ composite anode to mitigate electrochemical kinetic barriers, stabilize the electrode/electrolyte interface, and suppress electrolyte decomposition, thereby enabling improved long‐term cycling performance. The excellent electrochemical performance is not only attributed to the widely recognized improvements in electrode conductivity and suppression of Li dendrite growth brought about by Li alloys and Li solid solutions in the Li−Ag/Li_22_Sn_5_ electrode, but also to the low stress, strain, and their uniformity on the entire electrode during the charge/discharge processes. This is pivotal for securing structural stability in the Li metal anode and the full battery.

### 2.3 Full‐Cell Validation and Ah‐Level Pouch‐Cell Demonstration of the Li–Ag/Li_22_Sn_5_ Anode

**Figure 3:**
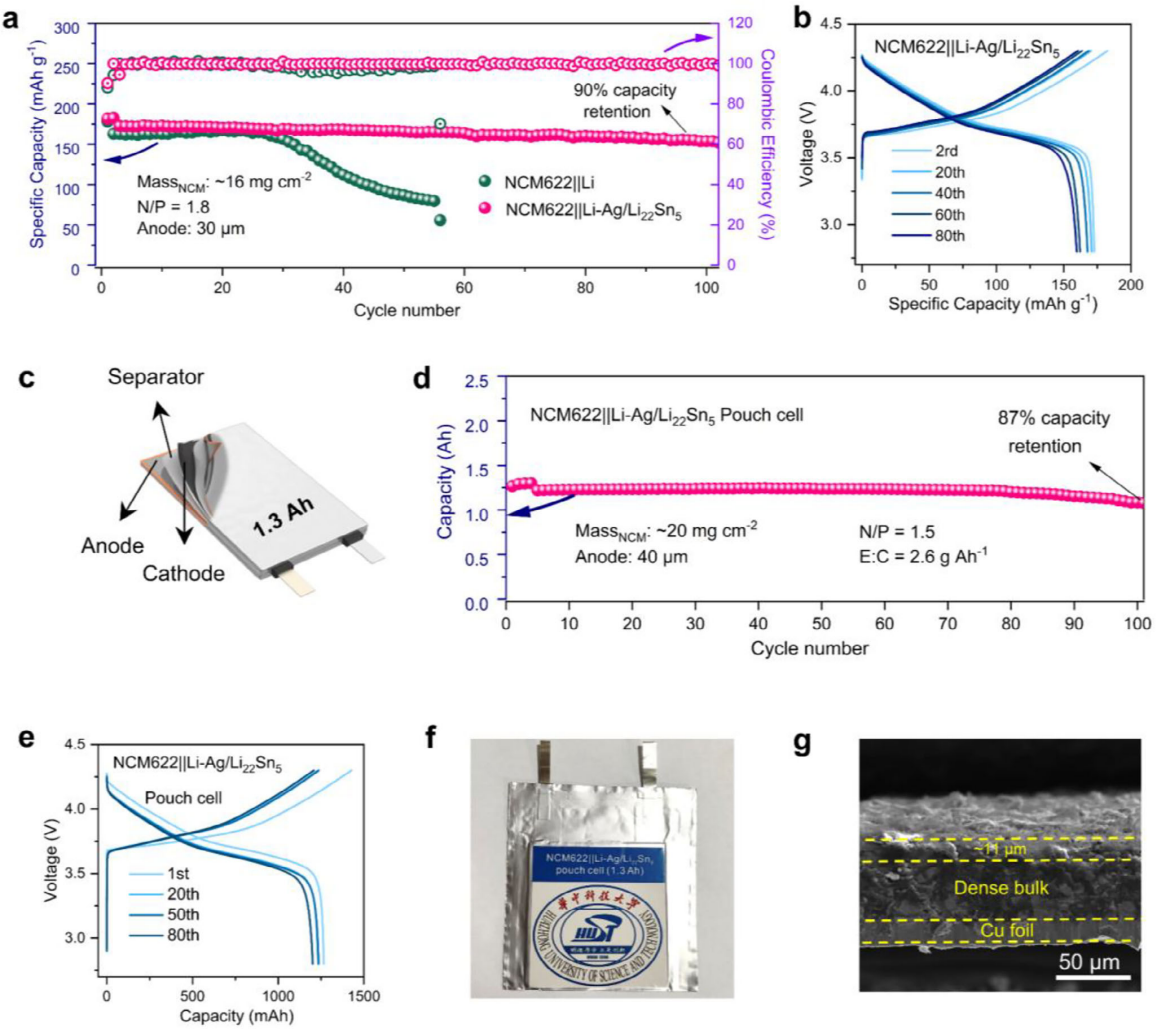
a) Cycling performance of NCM622||Li and NCM622||Li−Ag/Li_22_Sn_5_ full cells with 30 µm‐thick anodes. b) Corresponding galvanostatic charge/discharge profiles of the NCM622‖Li−Ag/Li_22_Sn_5_ cell. c) Schematic illustration of the Ah‐level laminated pouch‐cell configuration. d) Cycling performance of a laminated pouch cell comprising an NCM622 cathode and a 40 µm‐thick Li−Ag/Li_22_Sn_5_ foil anode (N/P=1.5, E/C =2.6 g Ah^−1^). e) Corresponding galvanostatic charge/discharge profiles of the pouch cell for the 1st, 20th, 50th and 80th cycles. f) Digital photograph of the Ah‐level laminated pouch cell. g) Cross‐sectional SEM image of the cycled Li−Ag/Li_22_Sn_5_ foil.

To further probe full‐cell behavior, NCM622||Li−Ag/Li_22_Sn_5_ and NCM622||Li cells were assembled. As depicted in **Figure** **3**a, the NCM622||Li−Ag/Li_22_Sn_5_ full cell exhibited an impressive initial areal capacity of 2.9 mAh cm^−2^ at 0.5 C and maintained ≈88% of its capacity over 100 cycles under a voltage window of 2.8–4.3 V with a low N/P ratio of ≈1.8. In contrast, although the NCM622||Li full cell delivered a comparable specific capacity during the first 30 cycles, it experienced a continuous decline thereafter, decaying to 50% of its initial capacity after 50 cycles. Galvanostatic charge/discharge profiles for both the NCM622||Li−Ag/Li_22_Sn_5_ and NCM622||Li cells are presented in Figures 3b and S18 (Supporting Information). Notably, the cell equipped with the Li−Ag/Li_22_Sn_5_ anode demonstrated stable capacity retention without any discernible increase in potential hysteresis during cycling, whereas the NCM622||Li cell exhibited a marked discharge‐plateau drop after 40 cycles. Elevated‐temperature testing highlights the composite's robustness. At 60 °C, NCM622||Li−Ag/Li_22_Sn_5_ cells retained 81% of their initial capacity after 100 cycles, whereas cells with Li anodes failed after only 40 cycles (Figure S19, Supporting Information). In tests employing LCO cathodes, cells featuring Li−Ag/Li_22_Sn_5_ anodes exhibited 97% capacity retention over 170 cycles (Figure S20, Supporting Information). Subsequently, the rate performance of LCO||Li−Ag/Li_22_Sn_5_ was implemented as shown in Figure S21 (Supporting Information), where the LCO||Li−Ag/Li_22_Sn_5_ delivered the high discharging capacities of ≈158.7, 144.3, 137.2, 121.4, and 157.1 mAh g^−1^ at 0.2C, 0.5C, 1C, 2C, 3C, 0.2C, respectively, higher than that of the LCO||Li full cell. The cyclic voltammetry curves of the LCO||Li−Ag/Li_22_Sn_5_ cell tested at 0.2 mV s^−1^ are shown in Figure S22 (Supporting Information). The Li−Ag/Li_22_Sn_5_ anodes were further evaluated in full cells paired with SPAN, which obtained long cycle life and stable capacity retention (Figure S23, Supporting Information).

To validate practical scalability, Ah‐level full pouch cells were assembled (Figure 3c). A 40 µm‐thick Li−Ag/Li_22_Sn_5_ foil with 8 × 8 cm was employed as the anode, while NCM622 cathode with a high mass loading of ≈20 mg cm^−2^ (3.6 mAh cm^−2^) was used as the cathode. Cell assembly was performed under stringent conditions, with an electrolyte‐to‐capacity (E/C) ratio of 2.6 g Ah^−1^ and a negative/positive capacity ratio of 1.5. As illustrated in Figure 3d,e, the Ah‐level pouch cell delivered a higher initial capacity (≈1.3 Ah) during the first four cycles, which can be attributed to the activation process conducted at a lower current rate of 0.1 C (where 1 C = 1.3 A). From the fifth cycle onward, the rate was increased to 0.2 C, leading to a slightly reduced but stable capacity (≈1.2 Ah). And it demonstrated stable cycling performance with a capacity retention of 87% and exhibited only a modest negative‐polarization drift. These outcomes underscore the contribution of the low‐strain, low‐stress composite anode to the stabilization of the electrochemical properties in practical Ah‐level full cells. Figure 3f presents a digital photograph of the Ah‐level laminated pouch‐cell, and Figure S24 (Supporting Information) and Figure 3 g exhibit cross‐sectional scanning electron microscopy (SEM) images of the Li−Ag/Li_22_Sn_5_ anode foil before and after cycling. The cycled foil shows a slight thickness increase of 7 µm compared to its pre‐cycling state, indicating its good structural stability. The foil electrodes displayed a planar surface and a dense bulk structure after 100 cycles, which demonstrates their capability to mitigate mechanically induced degradation during long‐term cycling.

### 2.4 Structural Evolution and Stability of the Li−Ag/Li_22_Sn_5_ Composite Anode

**Figure 4:**
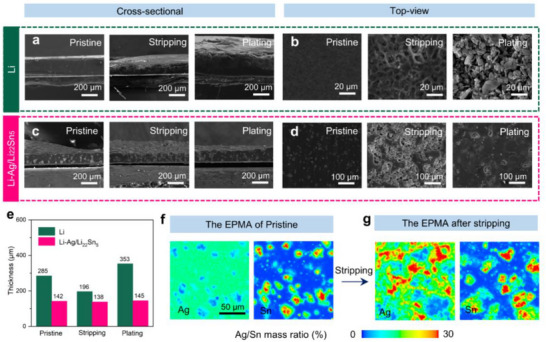
a,b) Cross‐sectional and top‐view SEM images of Li electrode at different states (pristine, after stripping 10 mAh cm^−2^ of Li, after plating 7 mAh cm^−2^ of Li). c,d) Cross‐sectional and top‐view SEM images of Li−Ag/Li_22_Sn_5_ electrode at different states (pristine, after stripping 10 mAh cm^−2^ of Li, after plating 7 mAh cm^−2^ of Li). e) Comparison of the thickness variations between the Li and Li−Ag/Li_22_Sn_5_ electrode across different states. f,g) Elemental mapping images for Ag and Sn, obtained by EPMA, of the Li−Ag/Li_22_Sn_5_ electrode in its initial state and following 3 mAh cm^−2^ Li stripping.

The Li_22_Sn_5_ framework combined with the Li−Ag solid solution promotes uniform and dense Li plating/stripping, thereby preserving the electrode structure during extended battery cycling. To elucidate the influence of skeleton configurations and the solid solution treatment on the electrochemical behavior of Li metal anodes, SEM observations were performed to examine the Li stripping and plating phenomena on different anode types. In **Figure** **4**a, it is apparent that during Li stripping from the Li foil, pronounced surface bulges with high curvature form readily, which are subsequently preferentially dissolved due to the “tip effect.”^[^ ^44^ ^]^ This process leads to the formation of numerous deep stripping pits, non‐uniform charge distribution, and irregular Li^+^ flux. Consequently, Li nucleates at the edges of these pits and grows into dendritic structures. During the plating stage, the Li anode experiences disordered Li deposition, resulting in loosely bound, porous Li plating products and significant, irregular electrode swelling. Figure 4b highlights the surface morphology of Li following Li plating, where a loosely arranged structure with distinct mossy Li dendrites is evident. In sharp contrast, the Li−Ag/Li_22_Sn_5_ electrode exhibited minimal thickness variation following Li stripping, as displayed in Figure 4c,d. Figure 4e illustrates the evolution of electrode thickness for both Li and Li−Ag/Li_22_Sn_5_ at the initial state, after stripping, and subsequent to lithiation. The Li electrode measured 285 µm initially, decreased to 196 µm after stripping 10 mAh cm^−2^ of Li, and swelled markedly to 353 µm following the plating of 7 mAh cm^−2^ of Li. In contrast, the Li−Ag/Li_22_Sn_5_ electrode exhibited only a slight variation, decreasing from 142 to 138 µm after stripping 10 mAh cm^−2^ of Li and showing a negligible thickness change to 145 µm after plating 7 mAh cm^−2^ of Li. This negligible variation underscores the high reversibility of Li stripping and plating, suggesting that the Li solid solution and Li alloy composite design effectively maintain structural integrity while suppressing volume swelling. The XRD patterns of the two states, stripping 10 mAh cm^−2^ of Li and plating 7 mAh cm^−2^ of Li, consistently exhibit distinct Li_22_Sn_5_ diffraction peaks (Figure S25, Supporting Information). The Li−Ag peak is markedly reduced after Li stripping, whereas it intensifies after Li plating. These results indicate that Li_22_Sn_5_ remains stable throughout the cycling of Li metal batteries, thereby contributing to the structural stability of the electrode.

Figures 4f,g, and S26 (Supporting Information) display top‐view electron probe microanalysis (EPMA) elemental mappings and back‐scattered electron (BSE) images of the Li−Ag/Li_22_Sn_5_ electrode in its initial state and after stripping 3 mAh cm^−2^ of Li. In the as‐prepared electrode, the Ag element was uniformly distributed throughout, whereas the Sn element appeared in irregular, micron‐scale domains. Following the dissolution of 3 mAh cm^−2^ of Li, the distributions of both Ag and Sn remained essentially unchanged, indicating that the Li−Ag/Li_22_Sn_5_ electrode maintained a stable composition and structure throughout the electrochemical process. This stability confirms that the Li_22_Sn_5_ alloy and Li−Ag solid solution effectively regulate the stripping and plating behavior of Li, thereby promoting long‐term cycle stability.

## 3 Conclusion

In summary, this work presents a composite Li metal anode strategy that integrates a Li solid solution with mixed ion‐electron conducting intermetallic frameworks to address the critical challenges of mechanical instability and parasitic reactions in Li metal batteries. By constructing a Li−Ag/Li_22_Sn_5_ composite anode, where Li_22_Sn_5_ provides a stable 3D skeleton for ion transport and mechanical support while the Li−Ag phase promotes uniform Li embedding, the system effectively maintains low interfacial strain and stress throughout prolonged cycling. Operando spatial stress analysis confirmed the structural integrity of the composite anode under dynamic electrochemical conditions. The practical relevance of this design was demonstrated in both coin and pouch cell configurations. When paired with a commercial NCM622 cathode, a comparison of the ΔP values during the 5th charging cycle (from 0% to 100% SOC) revealed that the NCM622||Li pouch cell exhibited a value of ≈20 kPa, which was 58% higher than the ≈12 kPa measured for the NCM622||Li−Ag/Li_22_Sn_5_ pouch cell. Furthermore, a 1.3 Ah laminated pouch cell using a 40 µm Li−Ag/Li_22_Sn_5_ anode exhibited excellent cycling stability, achieving 87% capacity retention over 100 cycles at a low N/P ratio of 1.5 and E/C ratio of 2.6 g Ah^−1^. This work provides a compelling framework for the structural regulation of Li deposition and interfacial stability, offering a scalable and practical pathway toward high‐performance Li metal batteries. The insights outlined here should translate to other solid solution/intermetallic pairs and guide next‐generation anode‐engineering strategies for safe, durable Li‐based energy storage.

## 4 Experimental Section

All experimental details are included in the Supporting Information.

## Conflict of Interest

The authors declare no conflict of interest.

## Author Contributions

J.F. and Y.S. contributed conceptualization, methodology. J.F., X.W. and X. D. performed investigation and writing. R.Z., C.L., T.D., Z.C., and H.S. handled data curation. S.L. and Y.O., managed editing. Y. S. performed supervision, writing–review and editing. All authors discussed and contributed to the results.

## Supporting information



Supporting Information

## Data Availability

The data that support the findings of this study are available from the corresponding author upon reasonable request.
