# Supplementary material for: Stress–Strain Engineering with Lithium Solid Solution/Lithium Alloy Composite Anodes for Stable Lithium‐Metal Batteries
Source: Adv Sci (Weinh). 2025 Sep 23;12(46):e15264. doi: 10.1002/advs.202515264 (PMC12697796; doi:10.1002/advs.202515264)
Supplement: Supplementary file 1 — Supporting Information [file ADVS-12-e15264-s001.docx]

Supporting Information

**Stress–Strain Engineering with Lithium Solid Solution/Lithium Alloy Composite Anodes for Stable Lithium-Metal Batteries**

*Junjie Fu, Xiancheng Wang, Xiangrui Duan, Zihe Chen, Tiancheng Dong, Hengtao Shen, Chunhao Li, Renming Zhan, Yangtao Ou, Shiyu Liu, Yongming Sun**

J. Fu, X. Wang, X. Duan, Z. Chen, T. Dong, H. Shen, C. Li, R. Zhan, Y. Ou, S. Liu, Prof. Y. Sun

Wuhan National Laboratory for Optoelectronics

Huazhong University of Science and Technology

Wuhan 430074, China

E-mail: yongmingsun@hust.edu.cn

Keywords: Stress-Strain, Li solid solution, Li Alloy, Li metal pouch cells, Operando spatial stress measurement

**Methods**

**Electrodes preparation:** Li-Ag/Li_22_Sn_5_ composite foils were fabricated via a sequential process that involved the production of a Li-Ag solution foil followed by the formation of the Li-Ag/Li_22_Sn_5_ composite foil under an inert atmosphere. Initially, Li metal was reacted with Ag metal at 250 °C to form a Li-Ag solid solution. Upon cooling, the Li-Ag solid solution was rolled into a foil. This foil was then stacked with a Sn foil of identical dimensions and subjected to repeated mechanical rolling. The mass ratio of Li, Ag, and Sn in the electrode was 6: 1: 3. The thickness of the resulting foil could be adjusted as required (Figure S2). The SEM images and the corresponding energy-dispersive X-ray spectroscopy (EDS) results were shown in Figure S3. The thickness of the foils could be seen in Figure S4 by cross-sectional SEM images. The content of the anode was tested by X-ray diffraction (XRD) in Figure S5. The transmission electron microscopy (TEM) (a) and EDS images of the Li-Ag/Li_22_Sn_5_ foil after the electrochemical stripping of Li were shown in Figure S6. And Figure S7 showed the capacity of Li-Ag/Li_22_Sn_5_ anodes. For comparison, Li/Li_22_Sn_5_ foils were fabricated by using a Li foil stacked with a Sn foil of identical dimensions and subjected to repeated mechanical rolling. The pure Li was molten, cooled, and calendared to prepare the Li electrode as a contrast. All operations were performed within an argon-filled glove box (O_2_/H_2_O < 0.1 ppm) to prevent surface oxidation. The NCM622, SPAN, and LCO cathodes were fabricated using slurry processes with respective compositions of 92:4:4, 85:7.5:7.5, and 94:3:3 (active material: carbon black: binder). Specifically, the NCM622 and LCO electrodes used PVDF as the binder, while the SPAN electrode utilized lithium polyacrylate (LiPAA) as the binder.

**Pouch cell assembly:** One piece of double-side NMC622 cathode (8 × 8 cm active area) and two pieces of Li-Ag/Li_22_Sn_5_ composite or Li foils anode were used to assemble NMC622||Li-Ag/Li_22_Sn_5_ and NMC622||Li laminated pouch cells to do the operando spatial pressure test. One piece of double-side SPAN cathode (8 × 8 cm active area) and two pieces of Li-Ag/Li_22_Sn_5_ composite or Li foils anode were used to assemble SPAN||Li-Ag/Li_22_Sn_5_ and SPAN||Li laminated pouch cells to do the operando spatial pressure test. Three pieces of double-side NMC622 cathode (8 × 8 cm active area) and six pieces of Li-Ag/Li_22_Sn_5_ foils anode were used to assembled the 1.3 Ah-level NMC622||Li-Ag/Li_22_Sn_5_ laminated pouch cells. All fabrication operations were conducted in an argon-filled glove box (< 1 ppm H_2_O and O_2_). The energy density containing specifications of the 1.3 Ah NMC622||Li-Ag/Li_22_Sn_5_ pouch cell is shown in Table S1.

**Operando spatial pressure test:** Operando spatial pressure monitoring was conducted using a force-sensor array film (G-PMS, SMiTSense) incorporating an array of precisely arranged force sensors. To enable real-time spatial pressure measurement, the fabricated pouch cell was placed between two plates of a clamping device pre-set to a defined initial external pressure (Figure S1). The force-sensor array film was deliberately positioned between the stainless-steel plate and the pouch cell. The electrochemical testing protocol for the pouch cell was synchronized with the operando pressure measurement to ensure coherent data acquisition. To calibrate the pressure sensors, the sensors are integrated with a computer running dedicated software for real-time monitoring. Regarding the sensor error margin, according to the specification parameters, the pressure sensing sheet (Model: SS-FPS-F606025) exhibits both consistency and repeatability errors of less than 5%, along with a durability of approximately 1 million cycles.

**Material characterizations:** In order to investigate the morphological and structural characteristics of the samples before and after cycling, SEM analyses were performed with a Nova NanoSEM 450 instrument. The electrode phases were determined via XRD using an Empyrean diffractometer, which operated with Cu-Kα1 radiation at an accelerating voltage of 40 kV and a current of 40 mA. Elemental composition and distribution were examined using EPMA with an 8050G system. Moreover, TEM, high-angle annular dark-field scanning TEM (HAADF-STEM), and EDX mapping via STEM-EDS were acquired using an FEI Talos F200X operating at 200 kV.

**Electrochemical tests:** In order to assess electrochemical performance, a 2032-type coin cell configuration was employed. The electrolyte comprised 1 M LiPF_6_ dissolved in ethylene carbonate (EC) and dimethyl carbonate (DEC) a 1:1 volumetric blend of, supplemented with 5 wt% fluoroethylene carbonate (FEC). Electrochemical impedance spectroscopy (EIS) measurements were performed in an open-circuit state using a VMP3 electrochemical workstation (Bio-Logic) over a frequency range of 100 mHz to 100 kHz. LiCoO_2_-based full cells were activated at 0.1 C (1C=170 mA g^−1^), then charged at 0.2 C and discharged at 0.5 C within a voltage window of 4.3-2.8 V. Similarly, NCM622-based full cells were activated at 0.1 C (with 1 C equivalent to 175 mA g⁻¹) and cycled at 0.5 C over the same voltage range. SPAN full cells were activated at 0.1 C (1 C = 700 mA g^−1^) for 1 cycle and then cycled at 0.5 C between 1.0–3.0 V. The NMC622 based pouch cells used to test operando spatial pressure were tested by galvanostatic charge/discharge mode at 0.2 C between 2.8 V and 4.3 V after two activation cycles at 0.1 *C* (1 C = 175 mA g^-1^). SPAN-based pouch cells for operando pressure were cycled at 0.3 C between 1.0–3.0 V after two activation cycles at 0.1 C. An Ah-level laminated NCM622||Li-Ag/Li_22_Sn_5_ pouch cell was activated at 0.1 C and subsequently cycled at 0.2 C. The theoretical specific capacity of the Li-Ag/Li_22_Sn_5_ anode for Li stripping/plating was calculated using the following expression:

Chemical formula: [(22 + x) Li + 5Sn + yAg→Li_x-_Ag_y_/Li_22_Sn_5_]

Theoretical capacity = 3860 mAh g^-1^ × Mass(xLi)/Mass(Li_x_-Ag_y_/Li_22_Sn_5_) = 2018 mAh g^-1^

If one assumes the complete delithiation of the Li_22_Sn_5_ intermetallic phase, a state that cannot be fully realized under practical cycling conditions in Li metal batteries, the maximum theoretical specific capacity of the composite anode is 2316 mAh g^-1^ including both the Li from Li-Ag and Li_22_Sn_5_, where the cut-off voltage for Li extraction should be higher than 0.4 V (Li extraction potential from Li_22_Sn_5_). Experimentally, as shown in Figure S7, the delithiation capacity measured with a cut-off voltage of 1.0 V is 2180 mAh g^-1^.


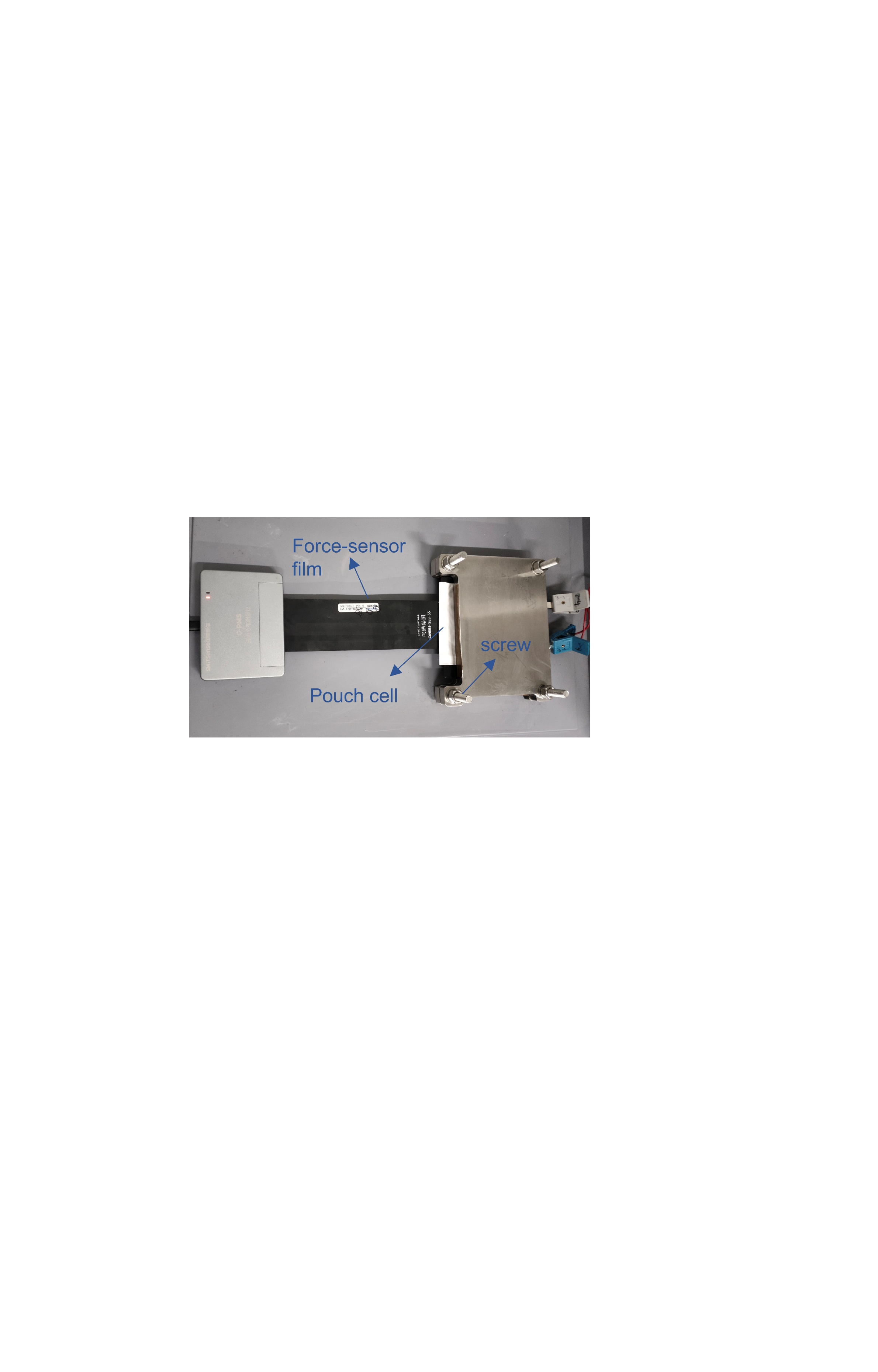


**Figure S1.** Optimal image of the operando spatial stress measurement setup.


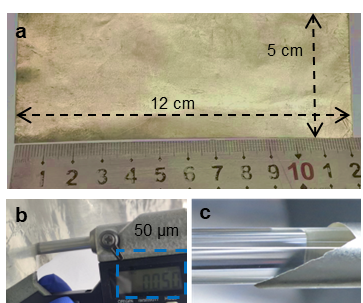


**Figure S2.** (a) Digital photos of the ultrathin Li-Ag/Li_22_Sn_5_ foil with size of 12 × 5 cm and thickness of 40 μm.


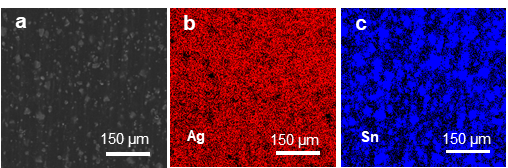


**Figure S3.** Top-view SEM images and associated EDS results for the Li-Ag/Li_22_Sn_5_ electrode.


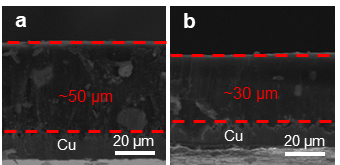


**Figure S4.** Cross-sectional SEM images of the thin Li-Ag/Li_22_Sn_5_ electrode with different thicknesses of (a) 50 μm, (b) 30 μm. Copper foil was employed to facilitate characterization.


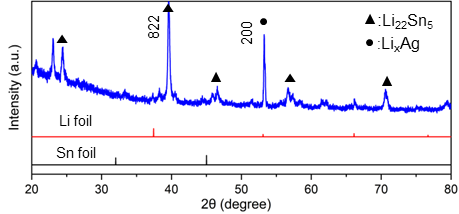


**Figure S5.** XRD of the Li-Ag/Li_22_Sn_5_ foil.


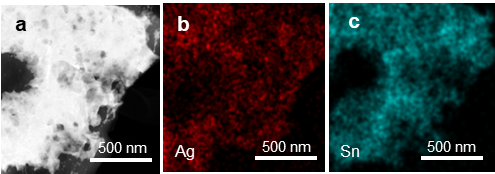


**Figure S6.** TEM (a) and EDS images of the Li-Ag/Li_22_Sn_5_ foil after the electrochemical stripping of metallic Li (b, c).


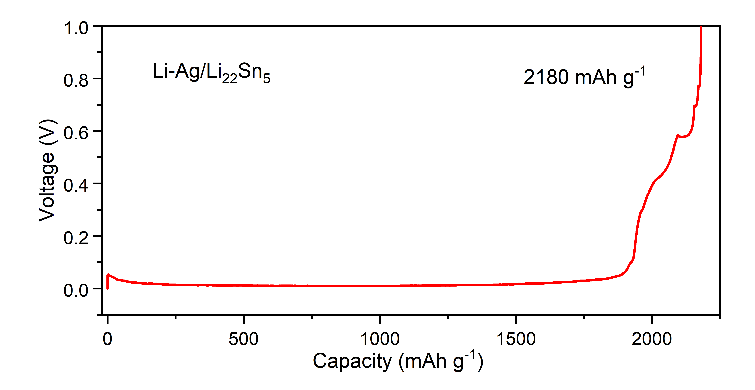


**Figure S7.** Voltage-capacity curves of Li stripping from Li-Ag/Li_22_Sn_5_ foil electrodes with the current density of 0.1 mA cm^-2^.


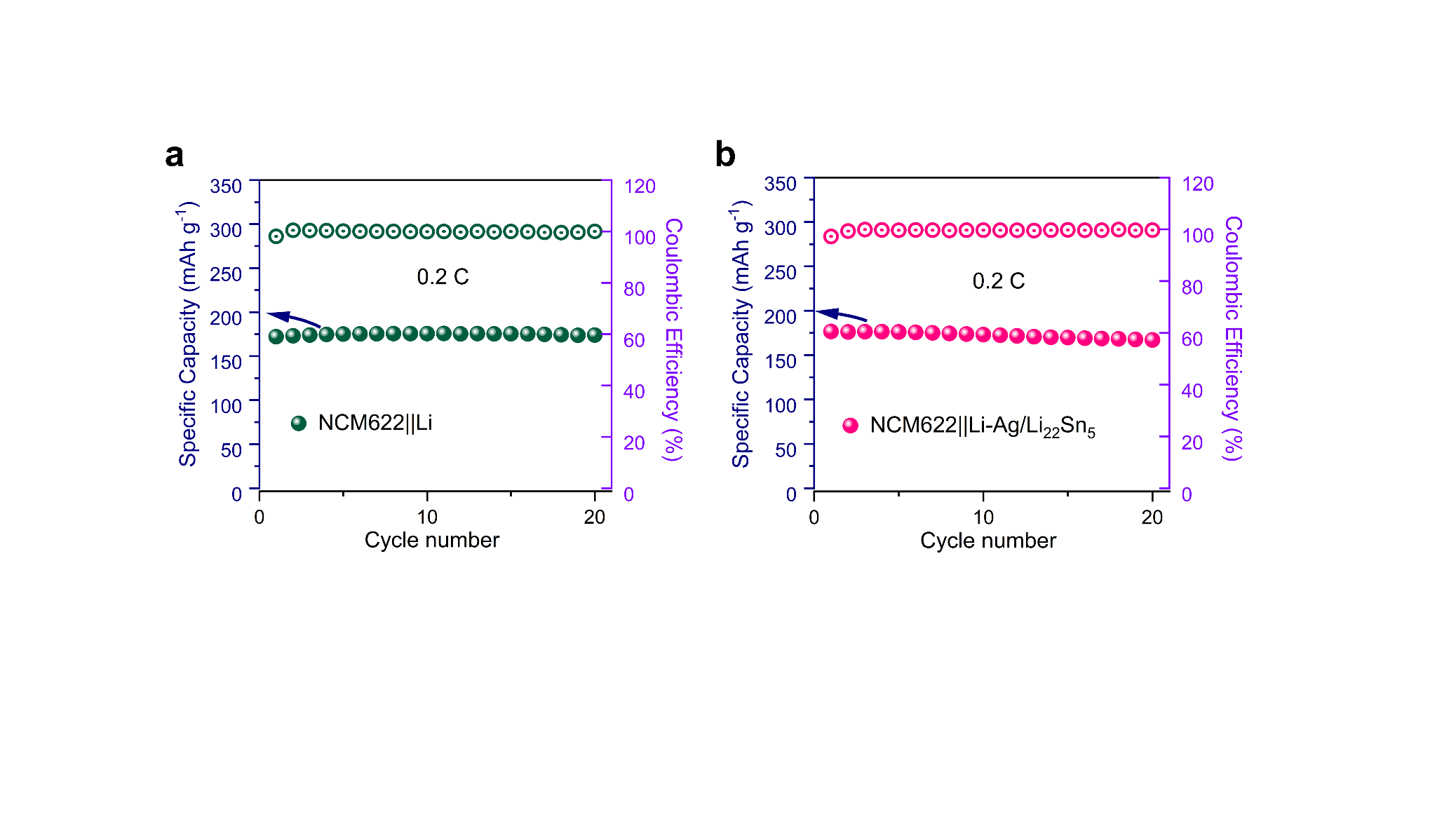


**Figure S8.** Cycling performance of NCM622||Li (a) and NCM622||Li-Ag/Li_22_Sn_5_ (b) pouch cells correspond to Figure 1.


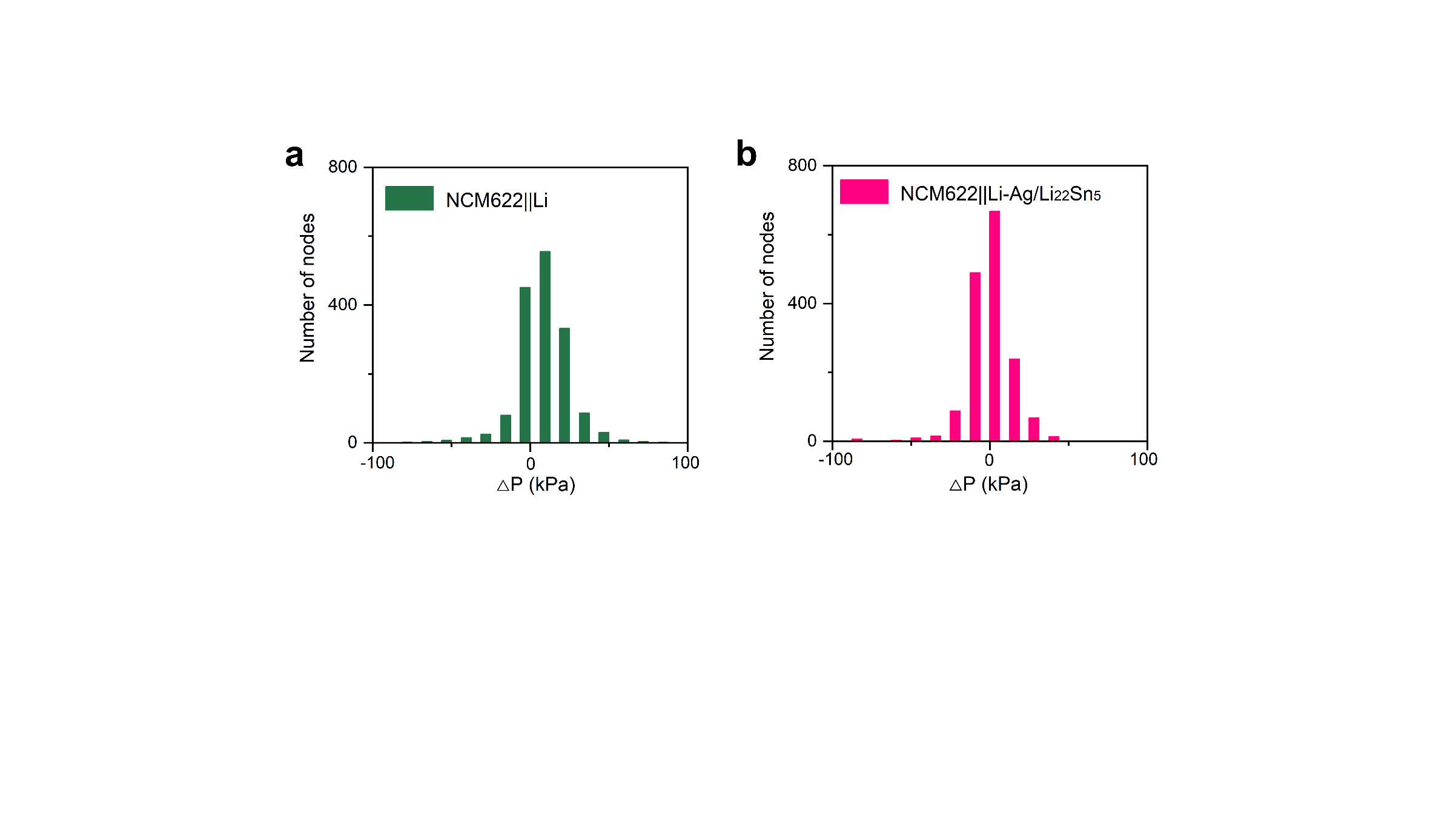


**Figure S9.** Distribution histogram of ΔP calculated from 1600 data points after charging at 0.2 C in the 5^th^ cycle of NCM622||Li (a) and NCM622||Li-Ag/Li_22_Sn_5_ (b) pouch cells.


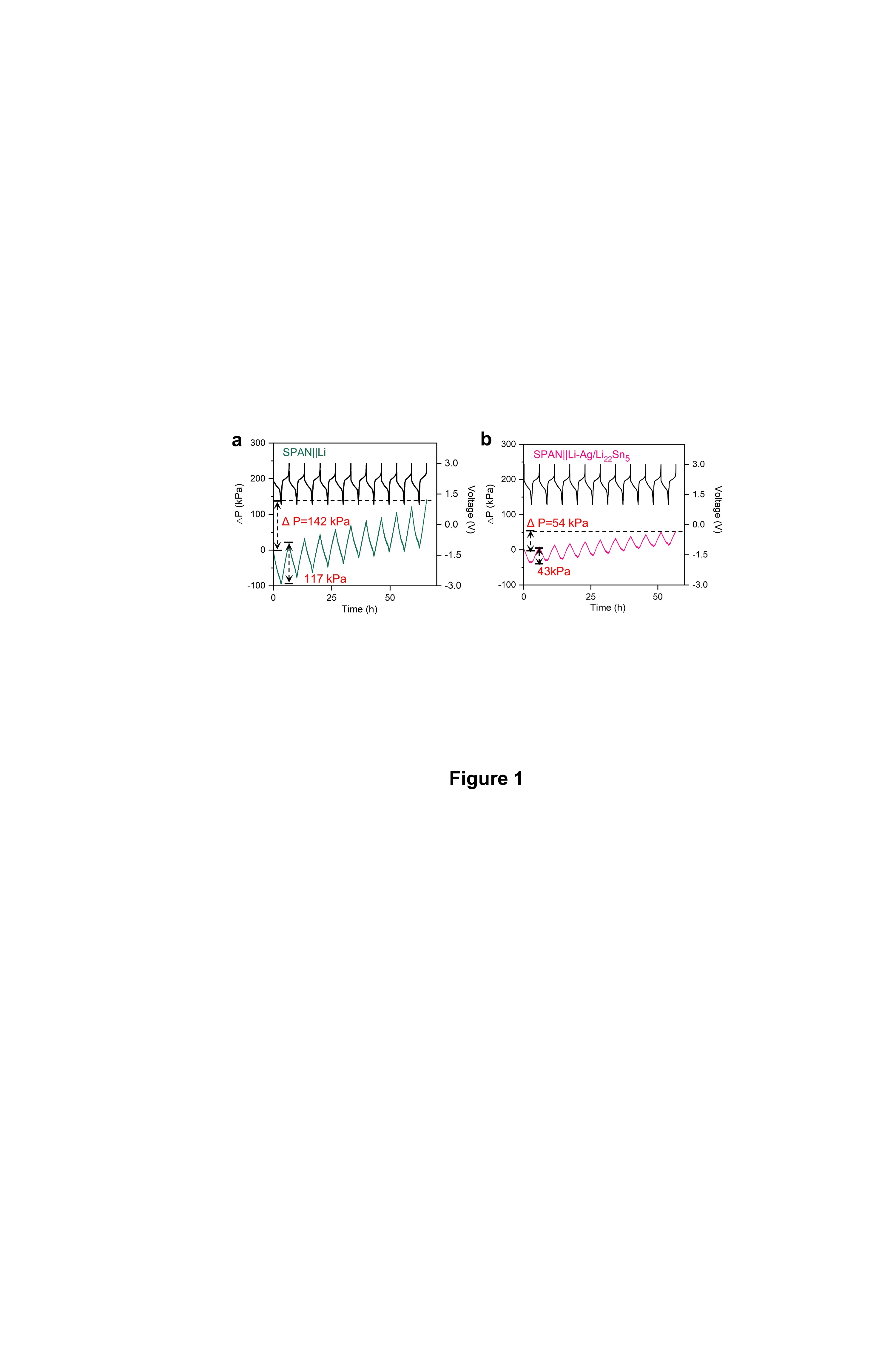


**Figure S10.** The evolution of self-generated pressures during charge/discharge for the first 10 cycles of SPAN||Li (a) and SPAN||Li-Ag/Li_22_Sn_5_ (b) pouch cells.


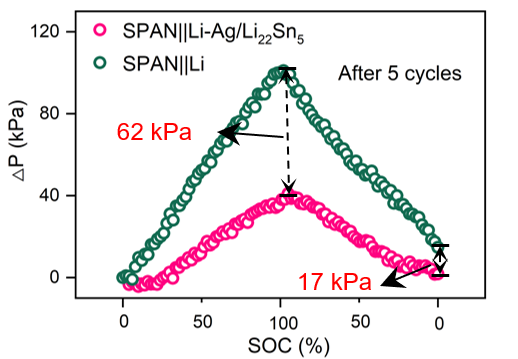


**Figure S11.** The evolution of pressure changes for the 5^th^ charge and 6^th^ discharge of SPAN||Li and SPAN||Li-Ag/Li_22_Sn_5_ pouch cells.


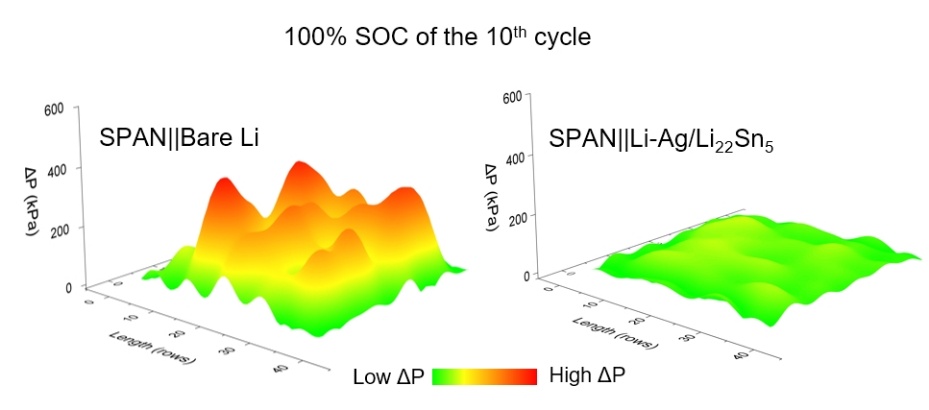


**Figure S12.** Three-dimensional of the pressure profiles after 10 cycles of SPAN||Li and SPAN||Li-Ag/Li_22_Sn_5_ pouch cells


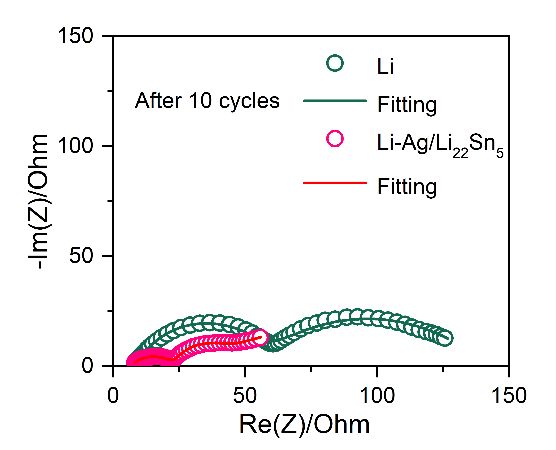


**Figure S13.** Nyquist plots of the Li and Li-Ag/Li_22_Sn_5_ symmetric cells after 10 cycles.





**Figure S14.** (a) The equivalent circuit employed for fitting Li symmetric cells, where R_s_, R_SEI_, and R_ct_ represent solution resistance, SEI film resistance and charge transfer resistance, respectively, while CPE_1_ and CPE_2_ denote constant phase elements. (b) The equivalent circuit employed for fitting Li-Ag/Li_22_Sn_5_ symmetric cells, where R_s_, R_SEI_, R_rl_ and R_ct_ represent solution resistance, SEI film resistance, reaction layer resistance and charge transfer resistance, respectively, while CPE_1_, CPE_2_ and CPE_3_ denote constant phase elements.


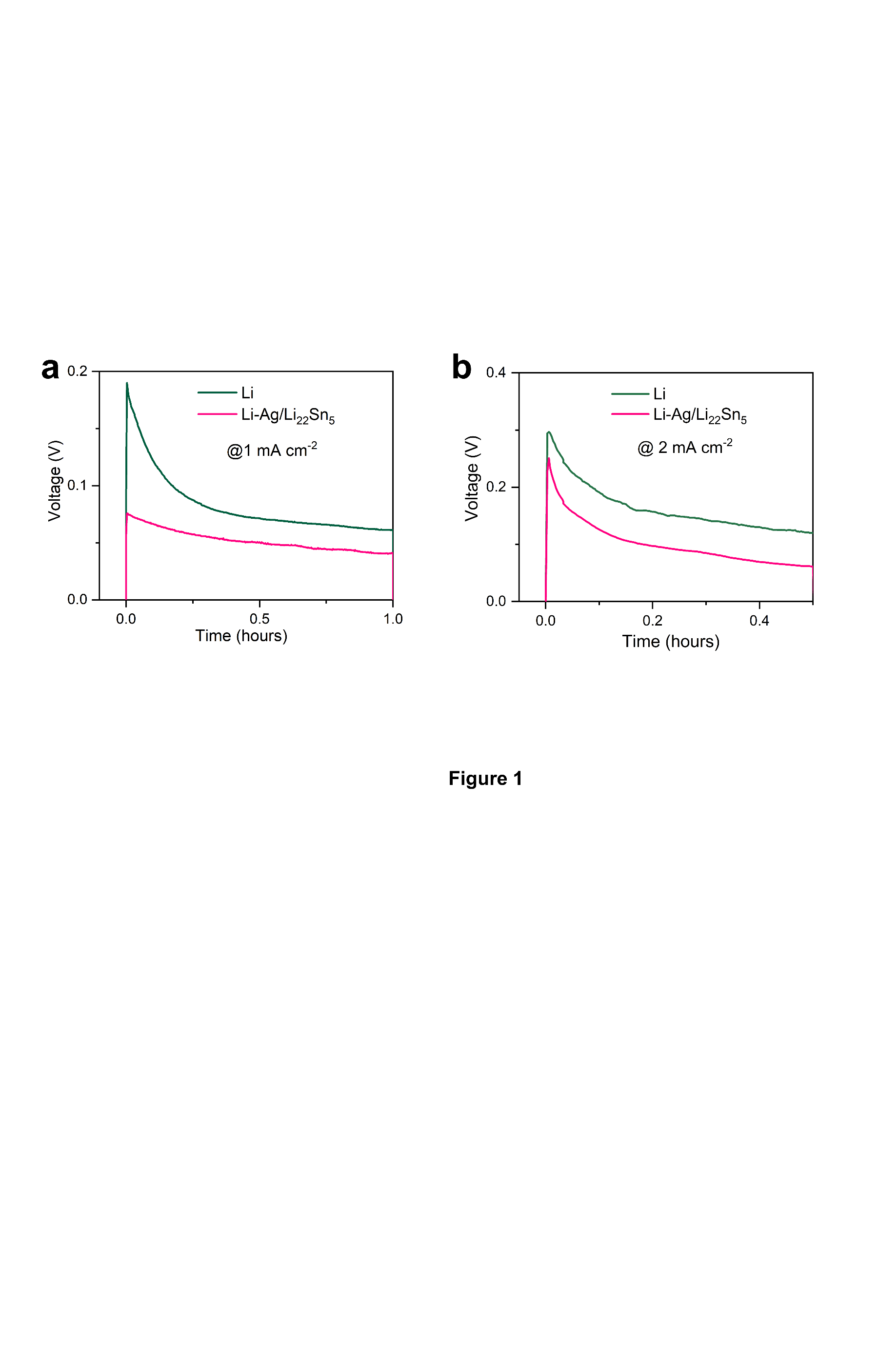


**Figure S15.** Nucleation overpotential curves for the electrochemical Li deposition on Li and Li-Ag/Li_22_Sn_5_ foil electrode at 1 mA cm^−2^ (a) and 2 mA cm^−2^ (b).

At both 1 mA cm^-2^ and 2 mA cm^-2^, the Li–Ag/Li_22_Sn_5_ electrode exhibits a markedly lower nucleation overpotential than pure Li, indicating facilitated Li nucleation and more uniform plating. The nucleation overpotential of the Li–Ag/Li_22_Sn_5_ electrode was 75 mV and 249 mV at 1 and 2 mA cm^-2^, respectively, substantially lower than the 191 mV and 321 mV measured for the Li electrode under the same conditions.


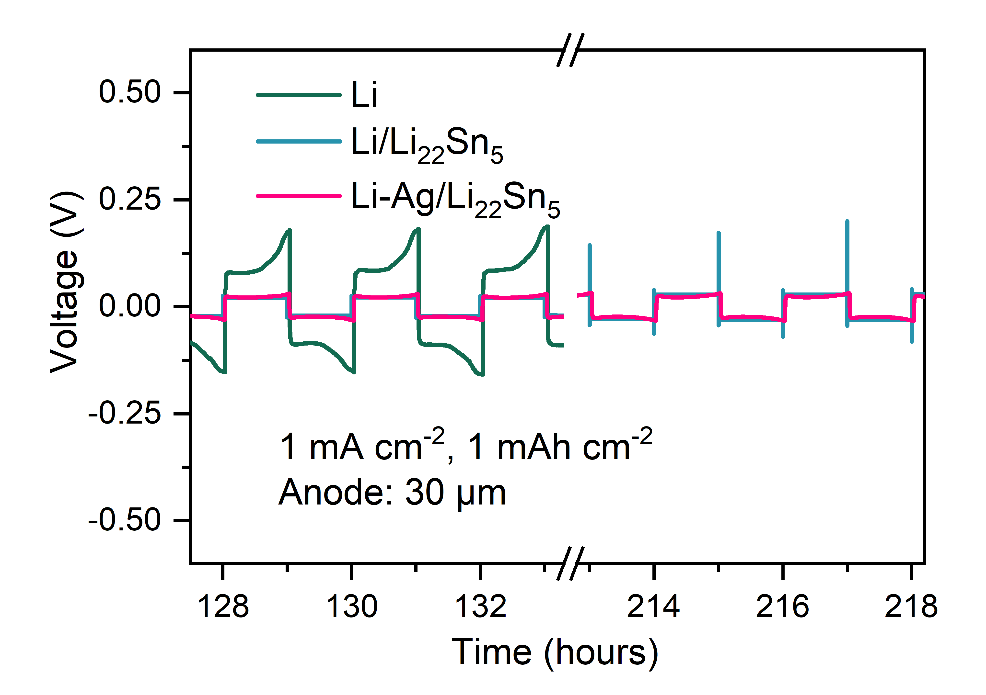


**Figure S16.** Magnified charge/discharge profiles for Li, Li/Li_22_Sn_5_, and Li-Ag/Li_22_Sn_5_ cells at selected cycles are presented.


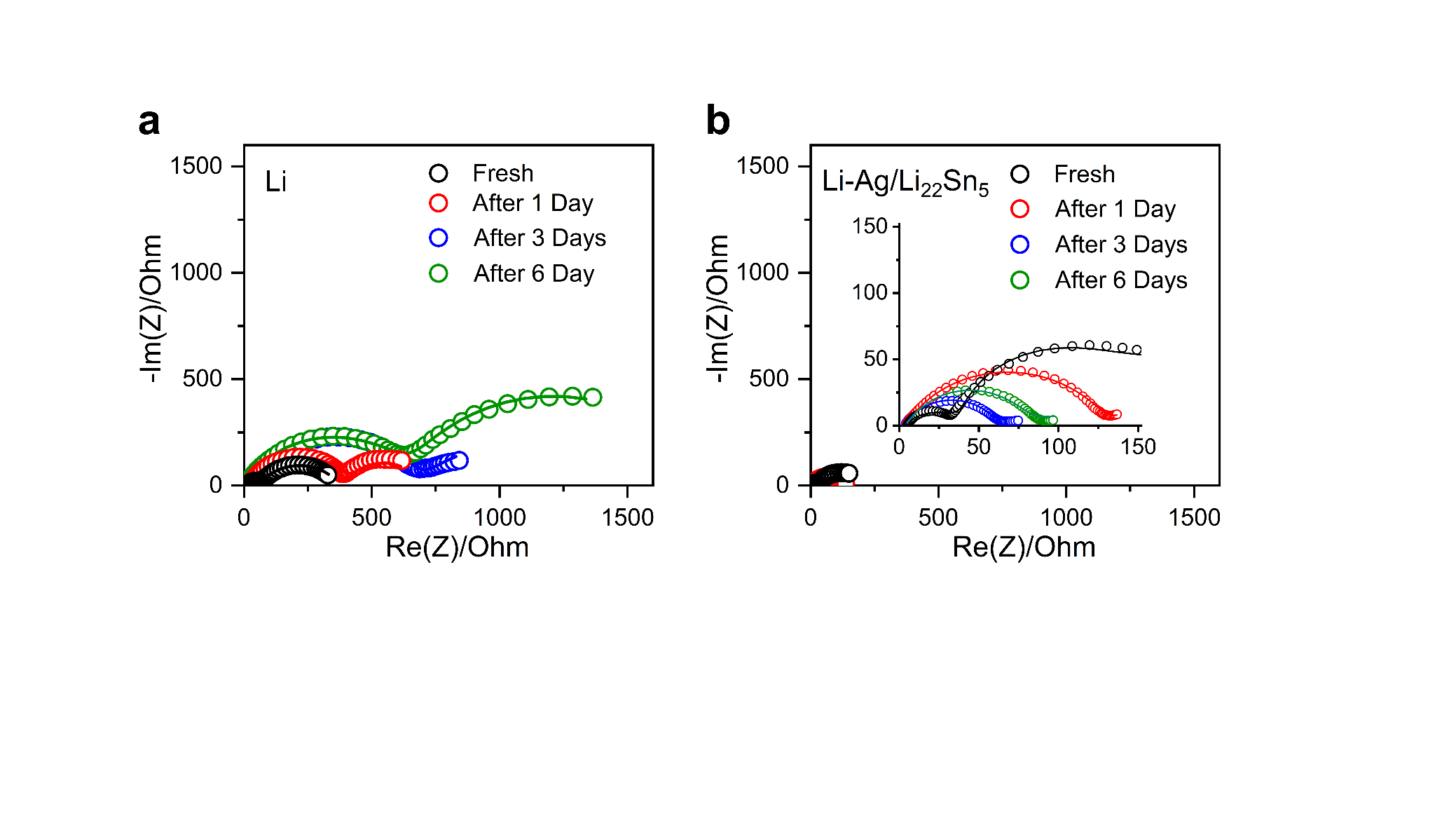


**Figure S17.** Nyquist plots of the Li and Li-Ag/Li_22_Sn_5_ symmetric cells after storage at 60 °C for different durations.


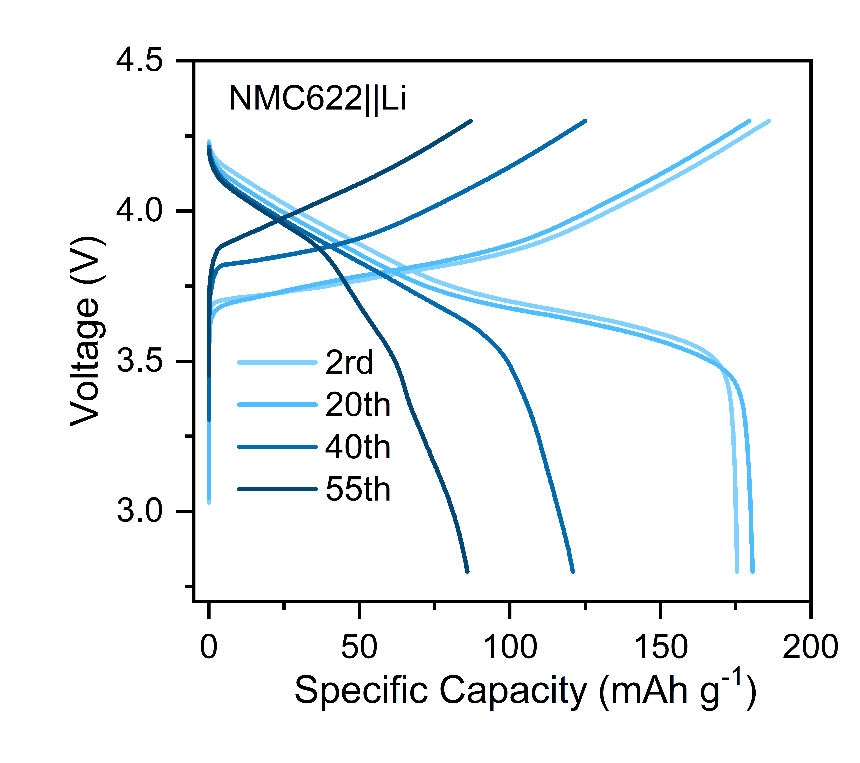


**Figure S18.** Voltage profiles of NCM622||Li full cell for selected cycles in Figure 3a.


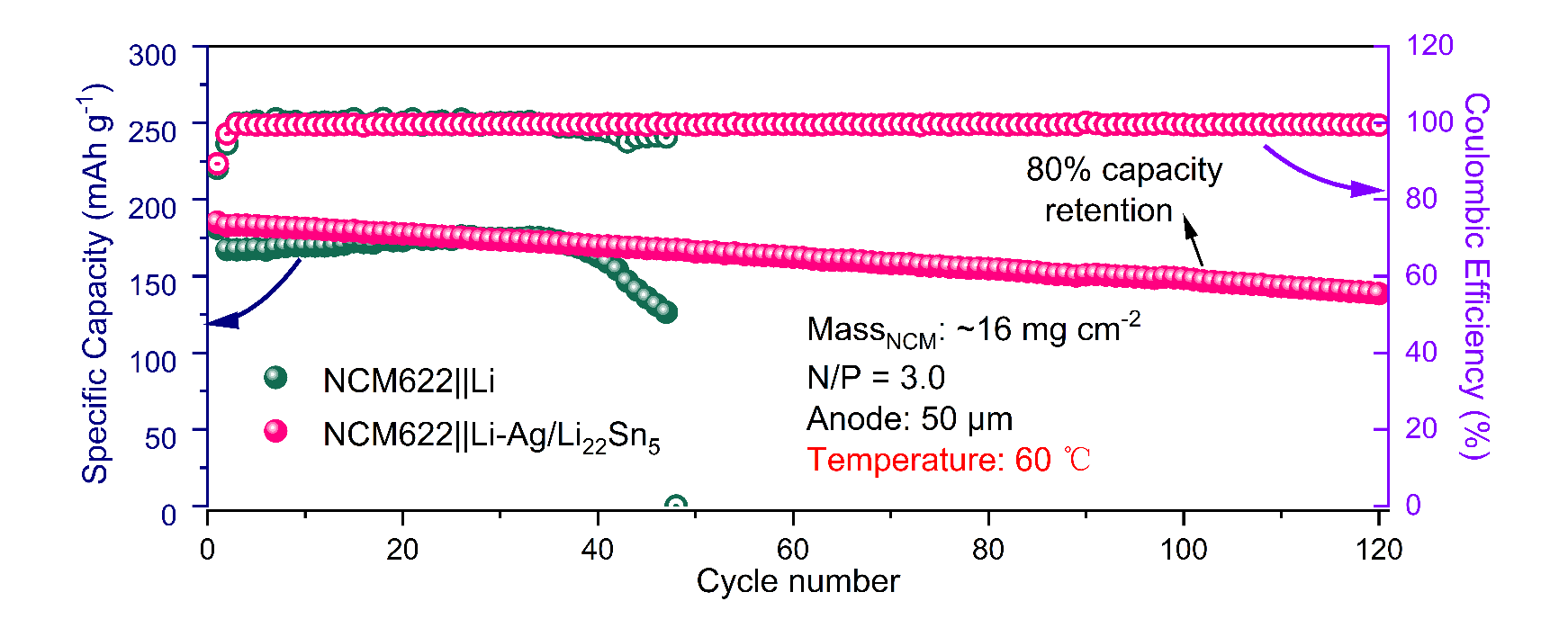


**Figure S19.** Cycling performance of NCM622||Li and NCM622||Li-Ag/Li_22_Sn_5_ full cells with anode thickness of 50 μm with low N/P ratio of 3 at high temperature of 60 ℃.


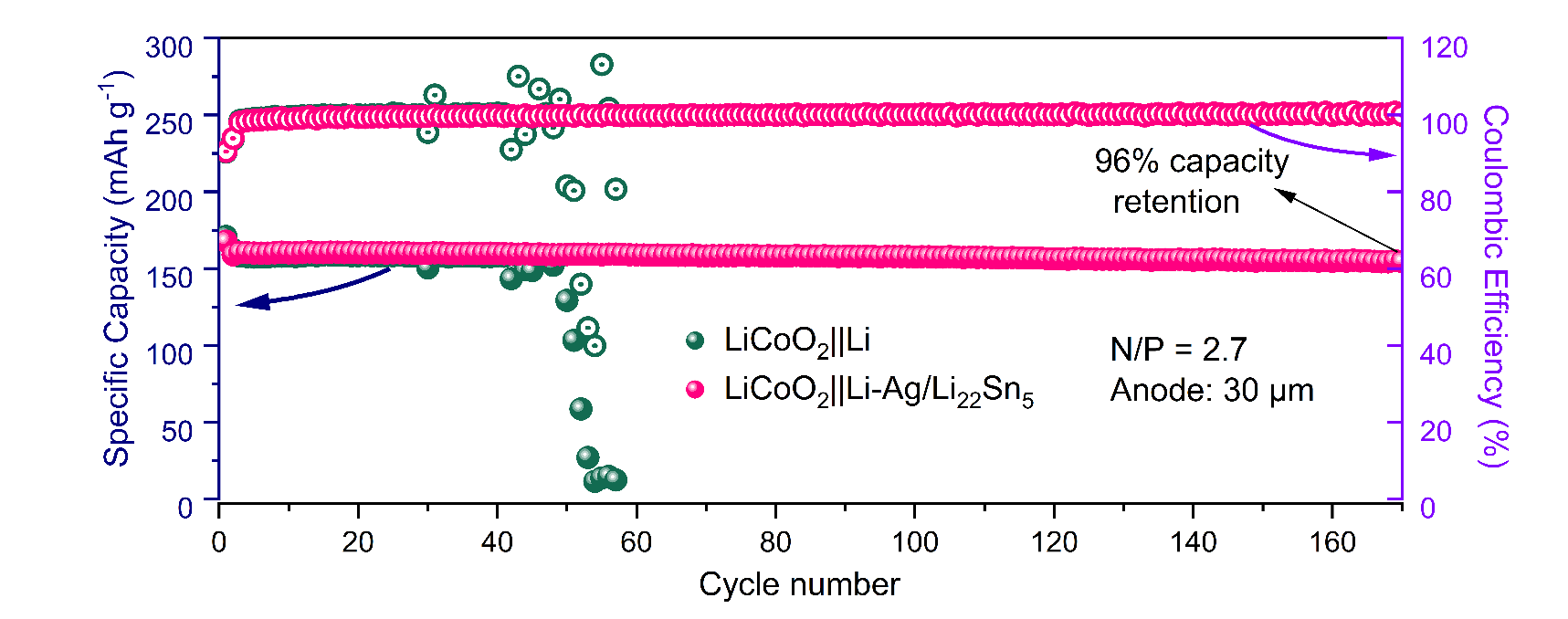


**Figure S20.** Cycling performance of LiCoO_2_||Li and LiCoO_2_||Li-Ag/Li_22_Sn_5_ full cells with anode thickness of 30 μm with low N/P ratio of 2.7.


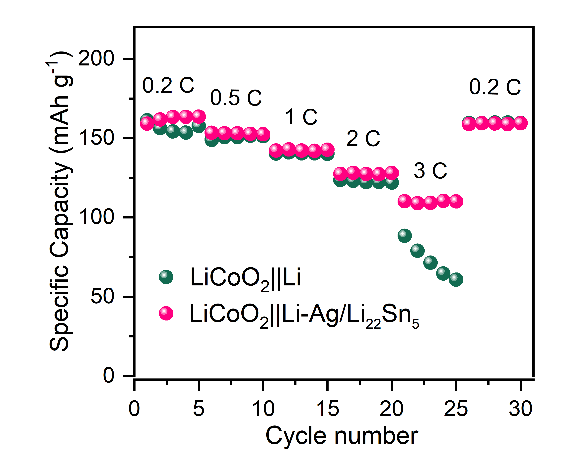


**Figure S21.** Rate capability of LiCoO_2_||Li and LiCoO_2_||Li-Ag/Li_22_Sn_5_ full cells.


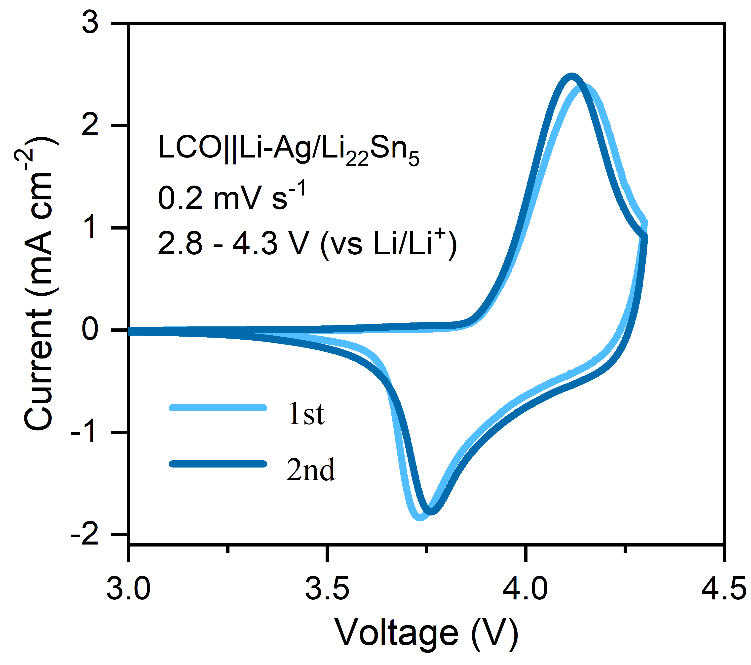


**Figure S22.** Cyclic voltammetry curves of the LCO||Li-Ag/Li_22_Sn_5_ cell tested at 0.2 mV s^-1^.


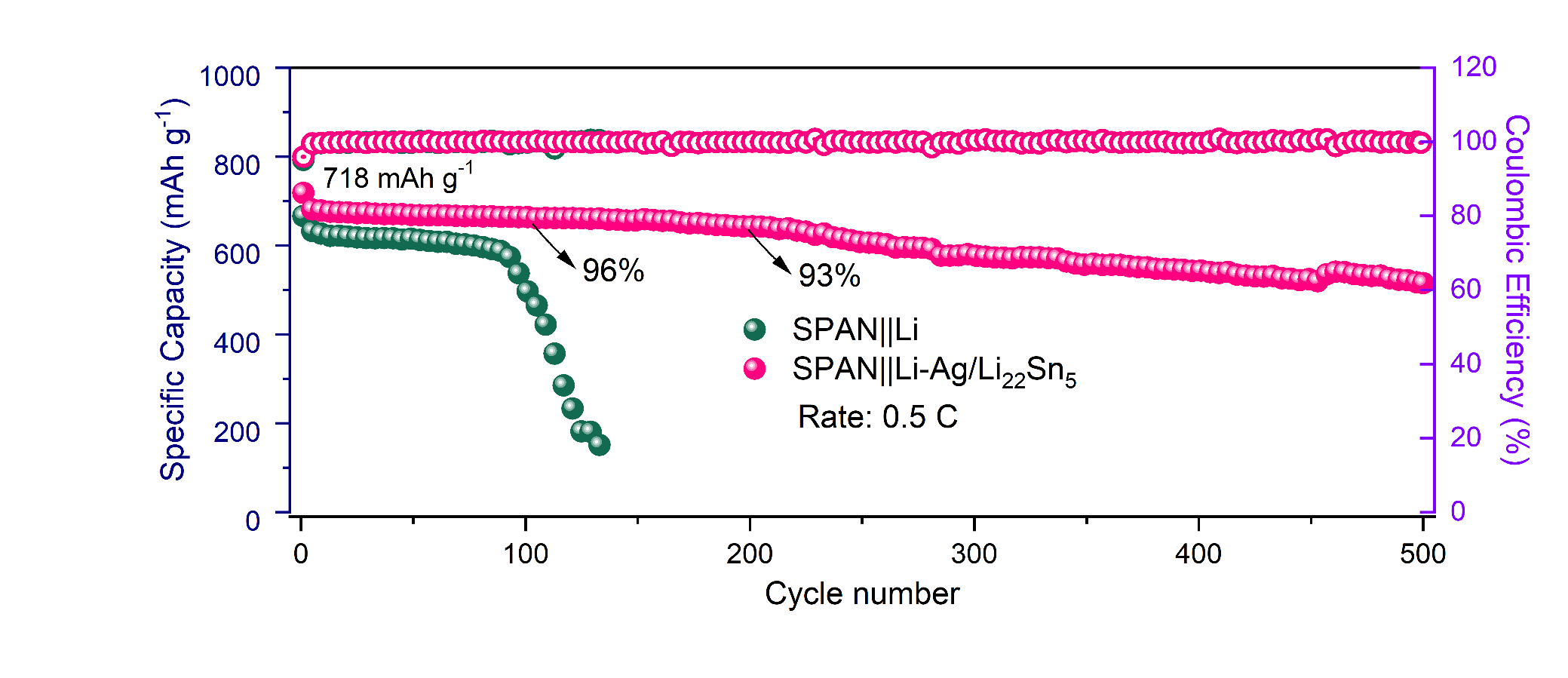


**Figure S23.** Cycling performance of SPAN||Li and SPAN||Li-Ag/Li_22_Sn_5_ full cells with anode thickness of 50 μm.


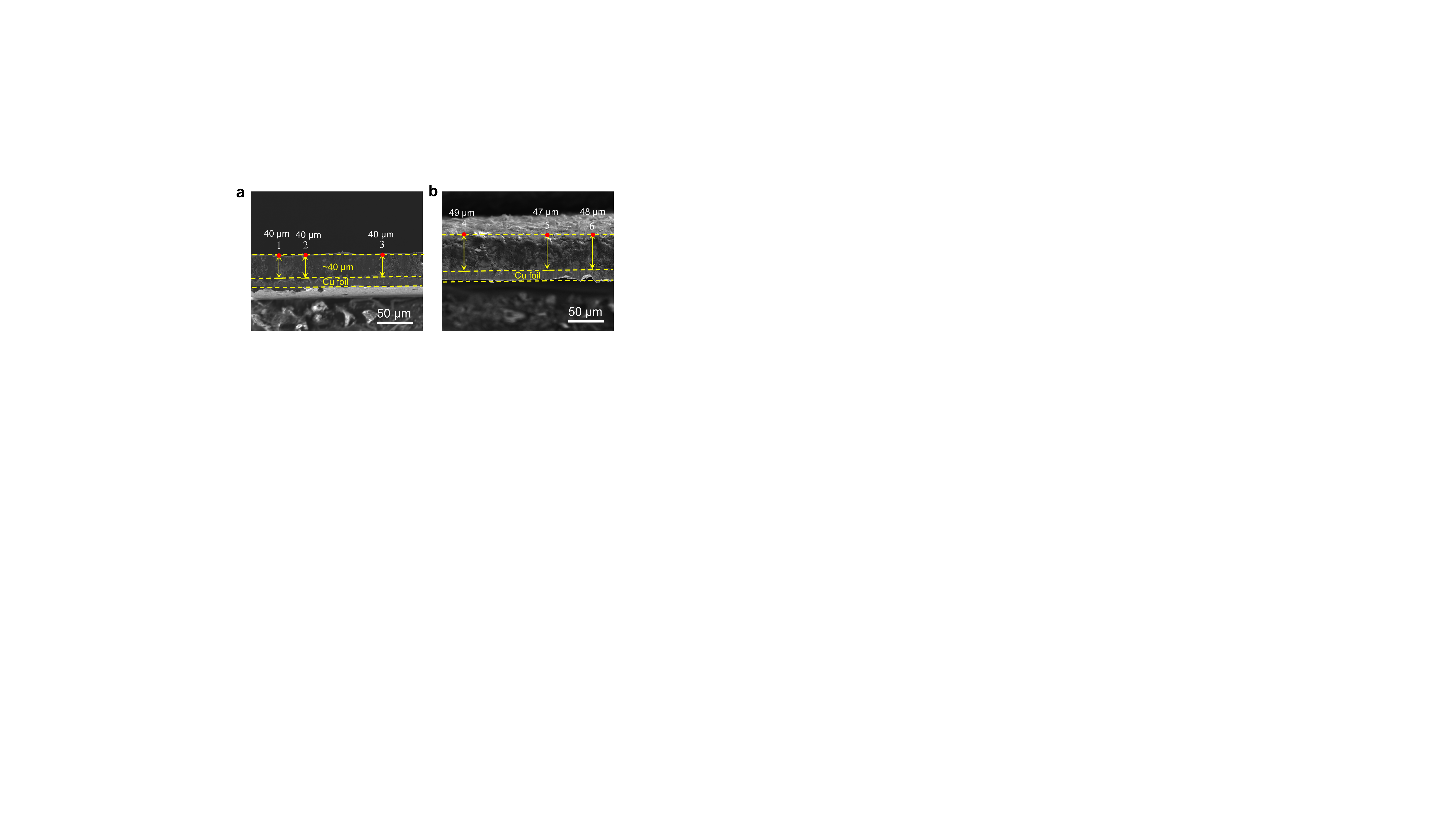


**Figure S24.** Thickness analysis of the Li-Ag/Li_22_Sn_5_ electrode before (a) and after (b) cycling in an Ah-level pouch cell with an NCM622 cathode.





**Figure S25.** XRD of the Li-Ag/Li_22_Sn_5_ foil after stripping 10 mAh cm^-2^ of Li and plating 7 mAh cm^-2^ of Li.


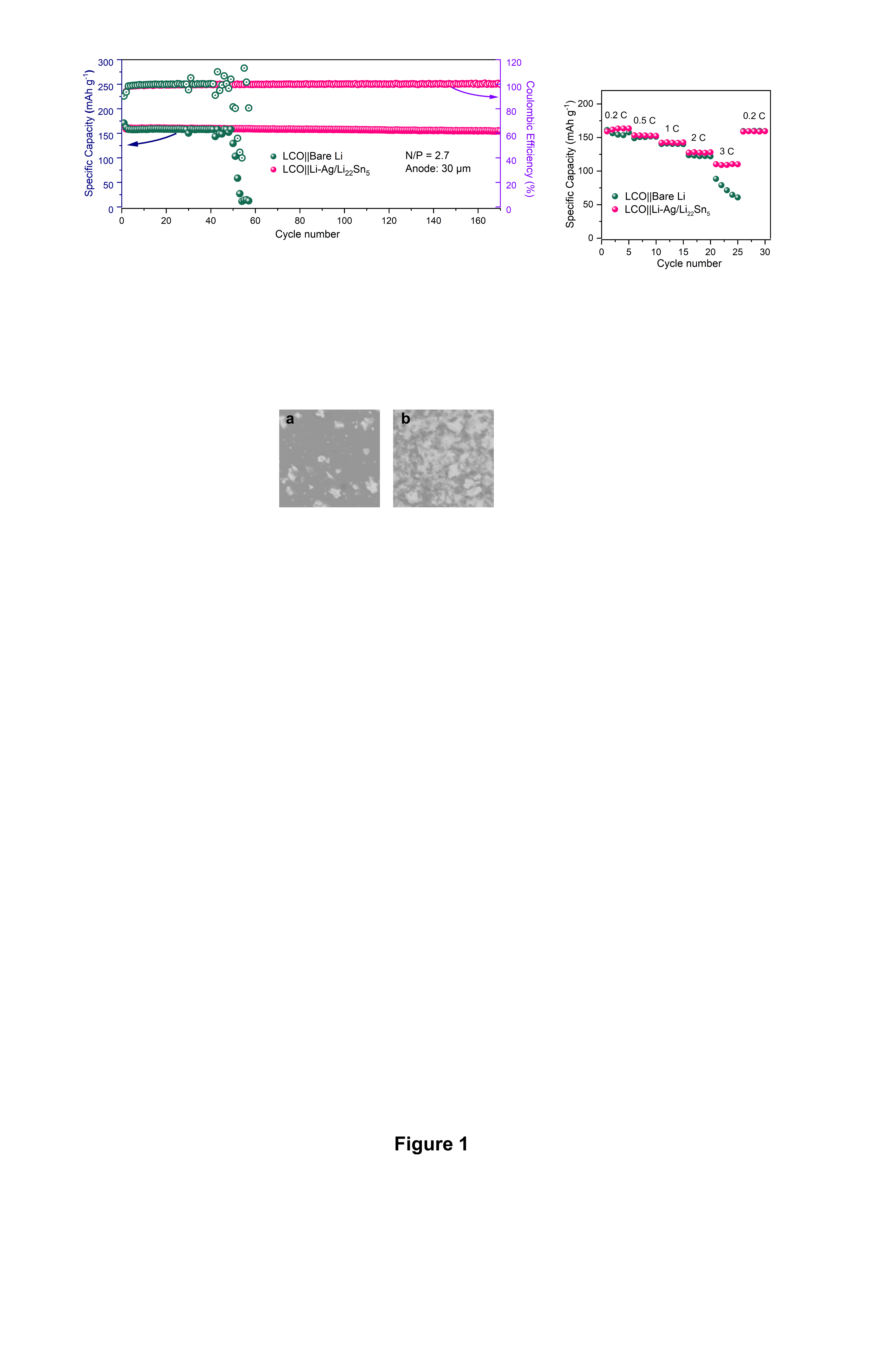


**Figure S26.** Back-scattered electron (BSE) images of Li-Ag/Li_22_Sn_5_ electrode at the state of pristine (a) and stripping 3 mAh cm^−2^ (b).


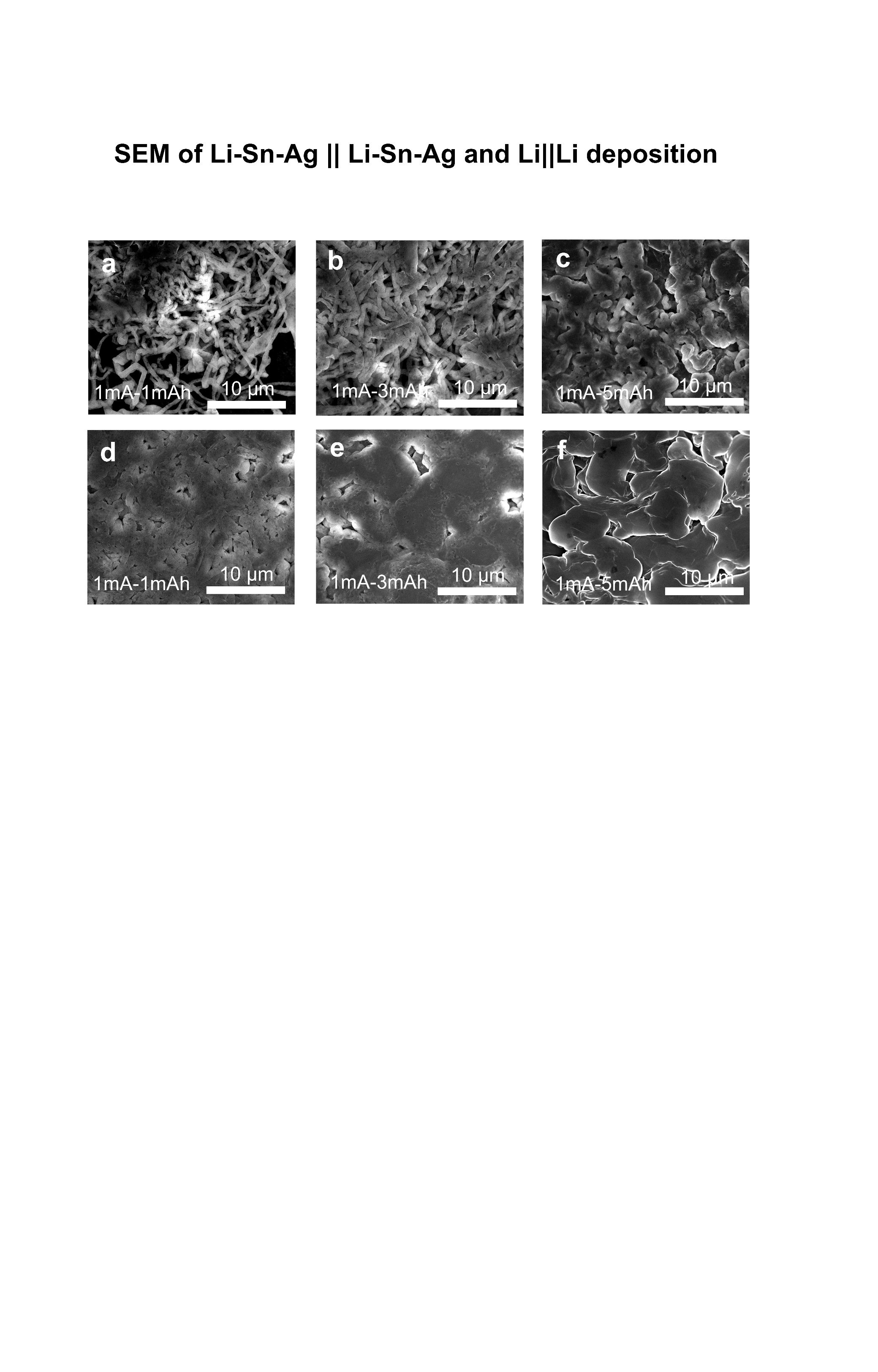


**Figure S27.** Top-view SEM images of Li (a, b and c) and Li-Ag/Li_22_Sn_5_ (d, e and f) electrodes after plating different (1 mAh cm^-2^, 3 mAh cm^−2^, 5 mAh cm^−2^) capacity Li metal.

**Table S1.** The specifications of the 1.3 Ah NCM622||Li-Ag/Li_22_Sn_5_ pouch cell.
